# Deciphering the pharmacological mechanisms of *Fraxini Cortex* for ulcerative colitis treatment based on network pharmacology and *in vivo* studies

**DOI:** 10.1186/s12906-023-03983-0

**Published:** 2023-05-09

**Authors:** Tianming Wang, Xuyang Su, Jing Peng, Xiaofen Tan, Guangshan Yang, Tengyue Zhang, Feng Chen, Changzhong Wang, Kelong Ma

**Affiliations:** 1 College of Integrated Chinese and Western Medicine (College of Life Science), Anhui University of Chinese Medicine, Hefei, 230012 People’s Republic of China; 2 Institute of Integrated Chinese and Western Medicine, Anhui Academy of Chinese Medicine, Hefei, 230012 People’s Republic of China; 3 Inflammation and Immune Mediated Diseases Laboratory of Anhui Province, School of Pharmacy, Anhui Medical University, Hefei, 230032 People’s Republic of China; 4 The First Affiliated Hospital of University of Science and Technology of China, Anhui Provincial Hospital, Hefei, 230001 People’s Republic of China

**Keywords:** Ulcerative colitis, *Fraxini cortex*, Network pharmacology, Molecular docking, In vivo studies

## Abstract

**Background:**

Ulcerative colitis (UC) is a common type of inflammatory bowel disease. Due to the elusive pathogenesis, safe and effective treatment strategies are still lacking. *Fraxini Cortex* (FC) has been widely used as a medicinal herb to treat some diseases. However, the pharmacological mechanisms of FC for UC treatment are still unclear.

**Methods:**

An integrated platform combining network pharmacology and experimental studies was introduced to decipher the mechanism of FC against UC. The active compounds, therapeutic targets, and the molecular mechanism of action were acquired by network pharmacology, and the interaction between the compounds and target proteins were verified by molecular docking. Dextran sulfate sodium (DSS)-induced colitis model was employed to assess the therapeutic effect of FC on UC, and validate the molecular mechanisms of action predicted by network pharmacology.

**Results:**

A total of 20 bioactive compounds were retrieved, and 115 targets were predicted by using the online databases. Ursolic acid, fraxetin, beta-sitosterol, and esculetin were identified as the main active compounds of FC against UC. PPI network analysis identified 28 FC-UC hub genes that were mainly enriched in the IL-17 signaling pathway, the TNF signaling pathway, and pathways in cancer. Molecular docking confirmed that the active compounds had high binding affinities to the predicted target proteins. GEO dataset analysis showed that these target genes were highly expressed in the UC clinical samples compared with that in the healthy controls. Experimental studies showed that FC alleviated DSS-induced colitis symptoms, reduced inflammatory cytokines release, and suppressed the expression levels of IL1β, COX2, MMP3, IL-17 and RORγt in colon tissues.

**Conclusion:**

FC exhibits anti-UC properties through regulating multi-targets and multi-pathways with multi-components. *In vivo* results demonstrated that FC alleviated DSS-induced colitis.

**Supplementary Information:**

The online version contains supplementary material available at 10.1186/s12906-023-03983-0.

## Background

Ulcerative colitis (UC) is a chronic and recurrent bowel disease, seriously impairs the quality of life, and aggravates the economic burden on patients [1]. In the last two decades, the incidence and prevalence of UC have continued to increase globally [2, 3]. The causes of UC still remain unclear, but the occurrence and progression of UC involves interactions among genetic, microbial, auto immune and environmental factors, making it challenging to develop effective drugs [3, 4]. Although several different types of drugs, such as 5-aminosalicylic acid, TNF-α blockers, and glucocorticoids, have been commonly used to treat UC [5, 6]. But these drugs are usually associated with high recurrence rates and adverse effects. Therefore, developing novel and safe strategies for UC treatment is urgently needed.

*Fraxini Cortex* (FC) is the dry barks of *Fraxinus rhynchophylla* Hance, *Fraxinus chinensis* Roxb, *Fraxinus szaboana* Lingelsh, or *Fraxinus stylosa* Lingelsh [7, 8]. It has been used as Chinese herbal medicine (THM) for various medical disorders due to its anti-inflammation [9, 10], anti-apoptosis [11] and antifibrotic effects [12]. Accumulated evidences have highlighted the beneficial roles or its ingredients of FC on prevention and treatment of UC [13]. In a previous study, the ethanol extract of FC was shown to exhibit anti-diarrheal function by affecting the transport of chloride ions in the rat intestinal epithelia [14]. Fraxinellone, a natural compound isolated from FC, was demonstrated to reduce weight loss and diarrhea in DSS-induced colitis mice, suppress the activities of myeloperoxidase and alkaline phosphatase, and increase the levels of glutathione in colitis tissues [15]. Besides, this compound also decreased the colonic levels of IL-1β, IL-6, IL-18 and TNF-α, and inhibited CD11b (+) macrophage infiltration [15]. Moreover, aesculin and aesculetin, another two natural compounds of FC, were proved to relieve the symptoms of DSS-induced colitis, restrain the secretion of TNF-α, IL-1β through inhibiting the activation of NF-κB and MAPKs pathway in colonic tissues and macrophages [16, 17]. However, the mechanisms of action of FC on UC treatment still remain elusive.

**Fig. 1:**
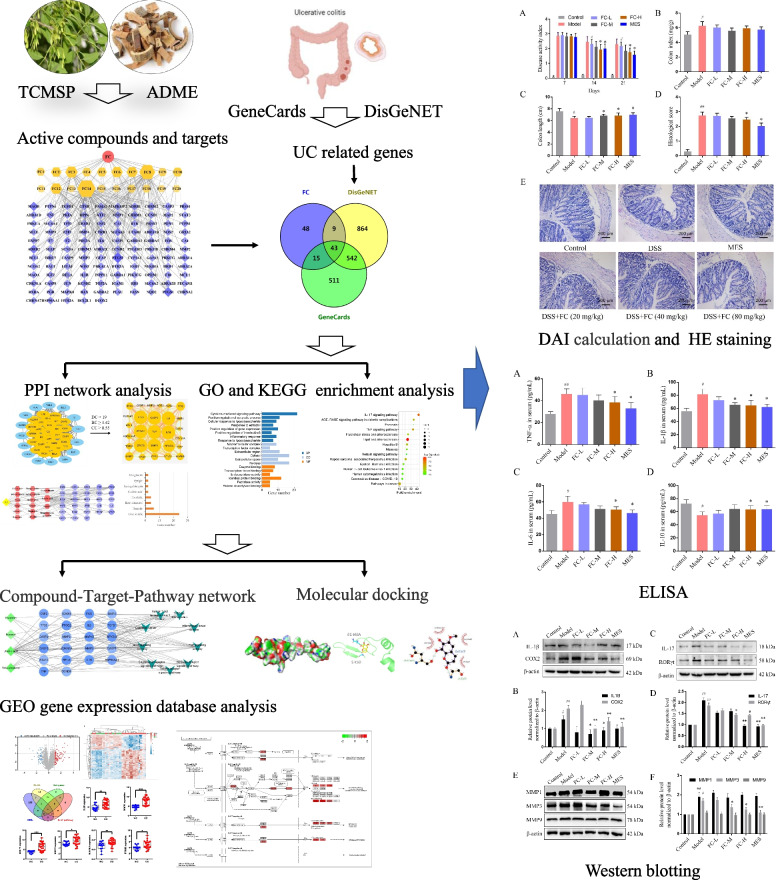
The framework of this study for exploring the pharmacological mechanisms of FC against UC

This study aimed to uncover the pharmacological mechanism of FC for UC treatment through a systems approach. Firstly, the active ingredients of FC were screened out based on the existing databases and pharmacokinetic characteristics. Then, the targets of the compounds, and the compound-target interactions were identified using comprehensive methods. The hub targets of FC against UC were obtained through the PPI network analysis. GO and KEGG pathway enrichment analyses were performed to predict the potential function of hub genes. Molecular docking and GEO microarray dataset were further used to identify the core targets, and in vivo studies were performed to assess the therapeutic effect of FC on DSS-induced colitis and validate the results of network pharmacology. The schematic overview of the process is summarized in Fig. 1.

## Methods

### FC active ingredients collection and screening of FC-UC targets

A Traditional Chinese Medicine Systems Pharmacology Database (TCMSP, version: 2.3) was used to collect the compounds contained in FC. The name and molecular structure of the compounds were verified by PubChem database. Predicted targets of all compounds were collected from Swiss Target Prediction and TCMSP databases. The official target names were standardized by the UniProt database (release 2022_02), and duplicates were excluded to obtain potential targets of FC against UC.

UC-related genes were collected from DisGeNET database (version 7.0) and GeneCards database (Version 5.8) with the keyword “ulcerative colitis, UC”, as our previous studies [18, 19]. The therapeutic targets of FC for UC were obtained by an intersection between FC potential targets and UC-related genes, and a Venn diagram was achieved by Venny 2.1.0 software.

### Protein-protein interaction (PPI) network construction

PPI network of the FC-UC targets was built via STRING database (version 11.5), and visualized by Cytoscape software (version 3.8.0). The topology analyses of the networks were conducted using CytoNCA plugin. The hub targets were obtained based on three topological parameters, including degree centrality (DC), betweenness centrality (BC) and closeness centrality (CC), according to network pharmacology evaluation method guidance [20].

### Gene ontology (GO) and Kyoto encyclopedia of genes and genomes (KEGG) pathway enrichment and analysis

GO function and KEGG pathway enrichment analyses of hub genes were conducted on the online platform of DAVID 2021(https://david.ncifcrf.gov/home.jsp) to uncover the detailed events of these genes involved in UC pathogenesis. The inclusion criteria was set as *p-*value < 0.05.

### Molecular docking

Four key pharmacodynamic molecules including ursolic acid, fraxetin, beta-sitosterol, and esculetin were selected to dock with 22 targets (degree score ≥ 5). The SDF format of compounds were obtained from the PubChem database (2021 update), and the target molecular structures of the proteins were derived from the RCSB protein database. The docking study was employed with a semi-flexible docking system, and conducted via AutoDock v4.2.6. PyMOL (version 2.5.4) was used to generate docking conformation, and analyze the lowest binding energy.

### Gene expression omnibus (GEO) database analysis

To study the expression levels of FC targeted UC genes, the expression profiling data from GSE22619 [21] and GSE37283 [22] were downloaded from the GEO database. The LIMMA package in the R software was used to filter the differentially expressed genes (DEGs) with the criteria of |Log2FC| > 1 and the *p*-value< 0.05. The expression distributions of DEGs in different tissue were showed as box plots. Statistical difference of two groups was analyzed by Wilcox test, and a *p*-value< 0.05 was considered statistically significant.

### *Fraxini cortex* extract preparation

*Fraxini cortex* extract was provided by Xi’an Quanao Biotech Co., Ltd. (Xi’an, China), and prepared as follows: *Fraxini Cortex* was chopped and grinded into small pieces, then extracted in 50% ethanol (v/v) with a ratio of 1/3 (g/mL) at 80 °C for 30 min twice. The extracts were centrifuged at 3500 rpm for 20 min at 4 °C, and concentrated and dried at 65 °C for 8 h to obtain powder. The contents of esculin and esculetin were 46.87 mg/g and 16.05 mg/g, respectively, according to the quality control report from the manufacturer. The power was redissolved in distilled water at various concentrations for animal experiments. The drug concentration was set based on a previous study [23] and our preliminary experimental results (Fig. S1).

### Mouse colitis model construction and drug treatment

C57BL/6 male mice (20 ~ 25 g) were purchased from Hangzhou Ziyuan Experimental Animal Corporation (Hangzhou, China, No. SCXK (Zhe) 2019–0004). DSS was purchased from MP Biomedicals Inc. (Santa Ana, CA, USA). MES was obtained from Ethypharm pharmaceutical company (Saint-Cloud Cedex, France). After adaptive feeding for 1 week, the mice were randomized into six groups of 10 each, control, model, FC low dosage (FC-L, 20 mg/kg), FC median dosage (FC-M, 40 mg/kg), FC high dosage (FC-H, 80 mg/kg), and mesalazine (MES) groups. The mice in model, FC, and MES group were given 3.0% (w/v) DSS for consecutive 7 days, respectively [24, 25]. Then, the mice in FC groups were administrated intragastrically with specified dose of FC for 14 days. The mice in MES group received 200 mg/kg MES, and the mice in the control and model groups were administered with the same volume of distilled water (Fig. S2). All animal experiments were performed with the prior approval from the Animal Ethics Committee of Anhui University of Chinese Medicine (AHUCM-mouse-2,020,037). At the end of the experiment, all animals were anesthetized with pentobarbital sodium (50 mg/kg, intraperitoneally) and sacrificed by cervical dislocation. Blood samples were collected by removing the left eyeball of the mice, centrifuged at 3000 rpm for 10 min to obtain serum, and kept at − 20 °C. The colon index and colon length of mice were measured as previous studies [18, 26, 27].

### Detection of cytokines in serum

Blood samples were collected and serum were isolated as in our previous reports [18, 26, 27]. Enzyme linked immunosorbent assay (ELISA) kits (MLBIO, Shanghai, China) were used to determine the serum concentrations of TNF-α (ml002095), IL-1β (ml301814), IL-6 (ml063159) and IL-10 (ml037873), according to the manufactures’ instructions.

### Histopathological analysis

The tissues were collected and fixed in 10% neutral-buffered formalin for over 24 hours. Then, the tissues were embedded in paraffin and cut into 4 μm thick sections for hematoxylin and eosin (H&E) staining. Histopathological score (HS) was scored using colon histopathology criteria [28].

### Western blot

Total proteins of colon tissues were extracted using Protein Prep Kit (Bio-Red, Hercules, CA, USA), and quantified by BCA Protein Assay Kits (Thermo Fisher Scientific, Waltham, MA, USA). Proteins were then resolved in a 10% SDS–polyacrylamide gel electrophoresis (SDS–PAGE) gel, and transferred onto a polyvinylidene fluoride (PVDF) membrane. After blocked with skimmed milk for 2 h, the membranes were incubated with primary antibodies overnight at 4 °C. The primary antibodies, IL-1β (AF510), MMP1 (DF6325), MPP3 (AF0217), MMP9 (AF5228), COX2 (AF7003), IL-17A (DF6127) and β-actin (AF7018) were obtained from Affinity Biosciences (Cincinnati, OH, USA), and anti-RORγt (14–6988-82) from Thermo Fisher Scientific (Waltham, MA, USA). After washing with PBST, the membranes were incubated in horseradish peroxidase-conjugated secondary antibodies (Elabscience, Texas, USA), and the bands were visualized with enhanced chemiluminescence (ECL) reagents. Signal intensities were quantified using Image J software (Bethesda, MD, USA).

### Statistical analysis

All data were presented as the mean ± standard deviation (SD), and the results were analyzed with SPSS 22.0 software (IBM, Armonk, NY, USA). Student’s *t* test was used for comparison of two groups, and one-way analysis of variance (ANOVA) was used for comparison among multiple groups. Statistical significance was defined as *p*-values less than 0.05.

## Results

### Screening of active compounds in FC and retrieval of FC-UC targets

**Fig. 2:**
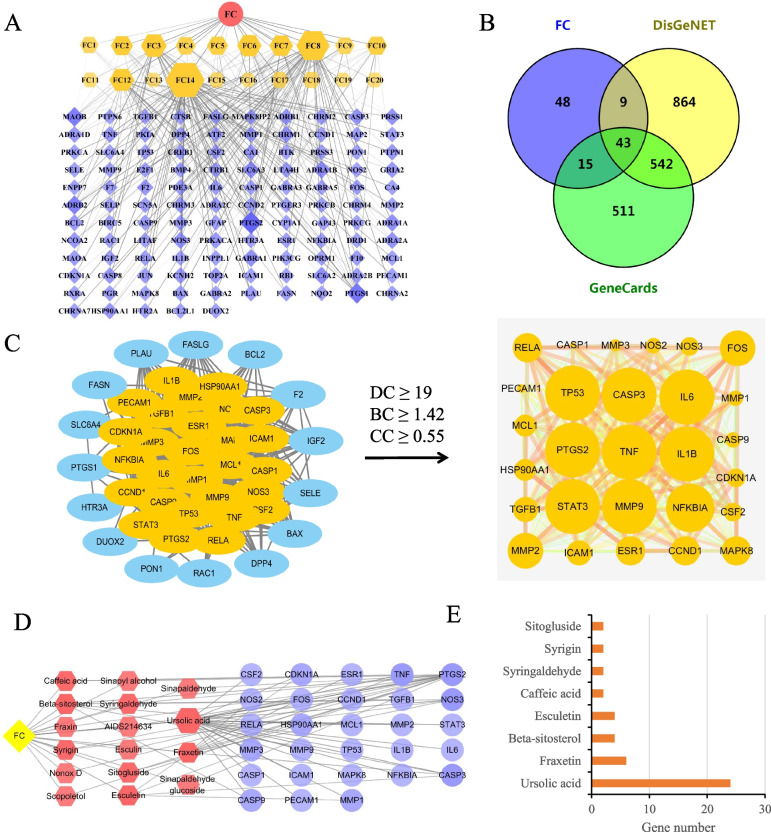
Active compounds and targets of FC against UC. **A** Compound-target (C–T) network. The nodes represent herb (red ellipse), active compounds (yellow hexagon), and target genes (blue diamond), respectively. **B** Venn diagram of FC targets and UC-related genes. **C** Topological screening for PPI network. **D** Core compound-target network. The yellow, red, and blue nodes represent herb, compounds, and targets, respectively. **E** The numbers of targets for the key compounds

A total of 20 FC active compounds were obtained from TCMSP database (Table S1). Although do not meet the criterion of ADME-related properties (oral bioavailability ≥30% and drug-likeness ≥0.18), several compounds including ursolic acid, fraxetin and esculetin were included in candidate components due to their poly-pharmacology features in FC. These 20 active compounds have 115 potential targets after remove the duplicates (Table S2). Compound-target network showed that ursolic acid (F14) possessed the most target genes (49 targets), followed by beta-sitosterol (F8) with 37 targets, caffeic acid (F3) with 21 targets and fraxetin (F12) with 19 target genes (Fig. 2A).

The UC-related genes were retrieved from the GeneCards database and DisGeNet database. Then, the FC potential targets were matched with these UC-related genes via the Venny software, and a total of 43 FC-UC common targets were acquired (Fig. 2B).

### Construction of PPI network

A network with 43 nodes (FC-UC common genes) and 459 edges was constructed basing on the results of the protein-protein interactions (PPI) analyzed by STRING database. According to topological analysis and the criteria of 2-fold median of DC, CC and BC, a core network with 28 nodes and 318 edges was generated (Fig. 2C). TP53, CASP3, IL6, PTGS2, TNF, IL1B, STAT3, MMP9 and NFKBIA have higher degree scores, which indicated that they might be the core targets of FC in UC treatment. Besides, these 28 key targets regulated by 16 compounds of FC (Fig. 2D). Among them, ursolic acid had the most targets, followed by fraxetin, beta-sitosterol, and esculetin, suggesting their potentially important roles in anti-UC function (Fig. 2E).

### GO and KEGG enrichment analysis

**Fig. 3:**
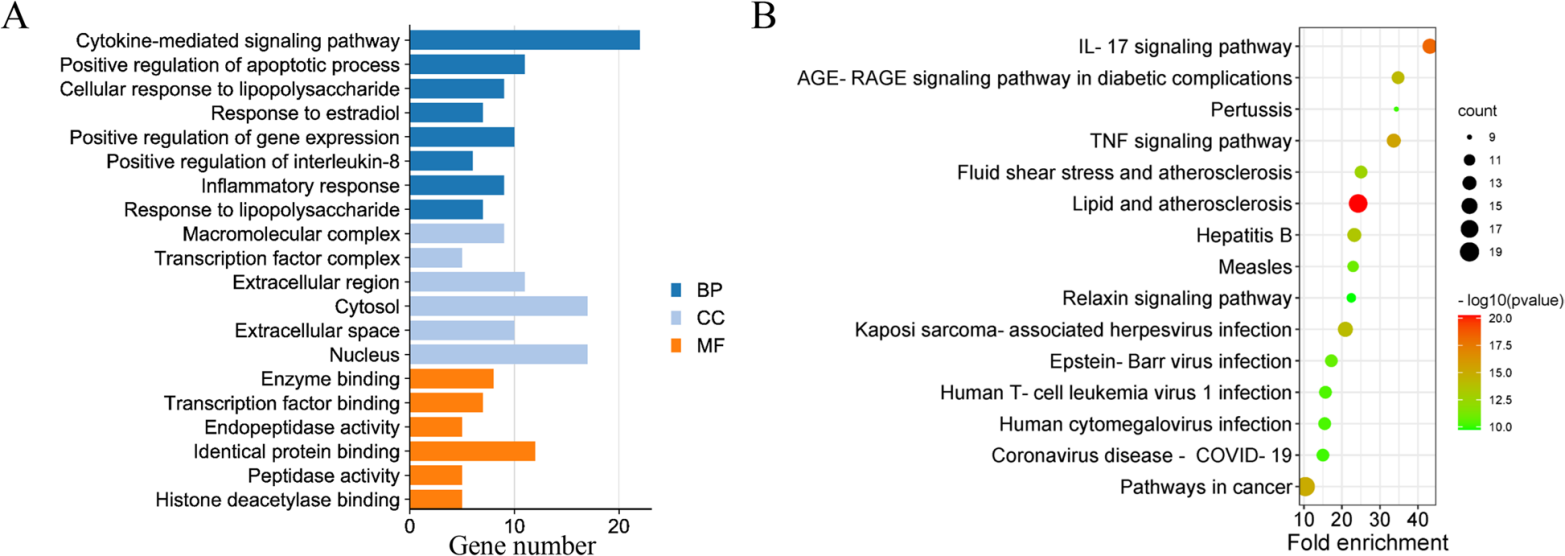
GO and KEGG pathway enrichment analysis of the hub genes. **A** GO enrichment of the top 8 BPs, 6 CCs and 6 MFs are displayed. **B** The top 15 KEGG pathway enrichments are displayed

GO enrichment analysis of 28 hub genes in the core PPI network totally got 269 GO items, including 214 items related to biological processes (BP), 15 items to cellular components (CC), and 40 items to molecular function (MF) (Table S3-S5). In the BP category, the genes were mainly enriched in cytokine-mediated signaling pathway, positive regulation of apoptotic process, and inflammatory response. In the CC, these genes were primarily enriched in cytosol and nucleus. In the MF, they enriched in enzyme binding and identical protein binding (Fig. 3A). KEGG pathway enrichment analysis showed that these hub genes highly participated in IL-17 signaling pathway, TNF signaling pathway and Pathways in cancer, indicating the multi-targets and multi-channels regulation of FC in UC treatment (Fig. 3B and Table S6).

### Compound-target-pathway network construction

**Fig. 4:**
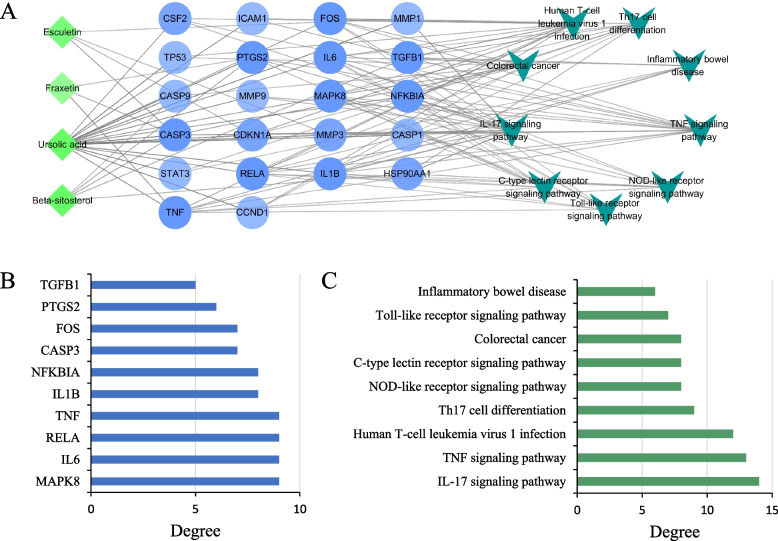
Compound-target-pathway network. **A** Compound-target-pathway network. **B** 10 major targets with top degree of interactions. **C** 9 key KEGG pathways related to FC against UC

A compound-target-pathway network was built by matching the nine key inflammatory and immune response-related pathways with the hub genes and FC components. This network consists of 35 nodes (4 compounds, 22 targets, 9 pathways) and 117 edges (Fig. 4A). Specifically, 10 major targets of FC against UC were identified with a higher degree≥5, i.e., MAPK8, IL6, RELA, TNF, IL1B, NFKBIA, CASP3, FOS, PTGS2 and TGFB1(Fig. 4B), and IL-17 signaling pathway was discerned with the highest degree (degree = 14) (Fig. 4C). The results provided a potential direction for molecular docking and future experimental validation.

### Molecular docking analysis

**Fig. 5:**
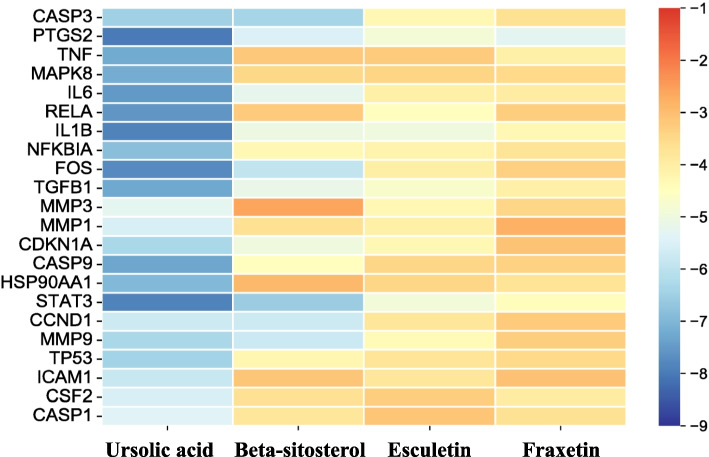
Affinity heat map. The map colors represent binding energy values (kcal/mol) increasing from blue to red

**Fig. 6:**
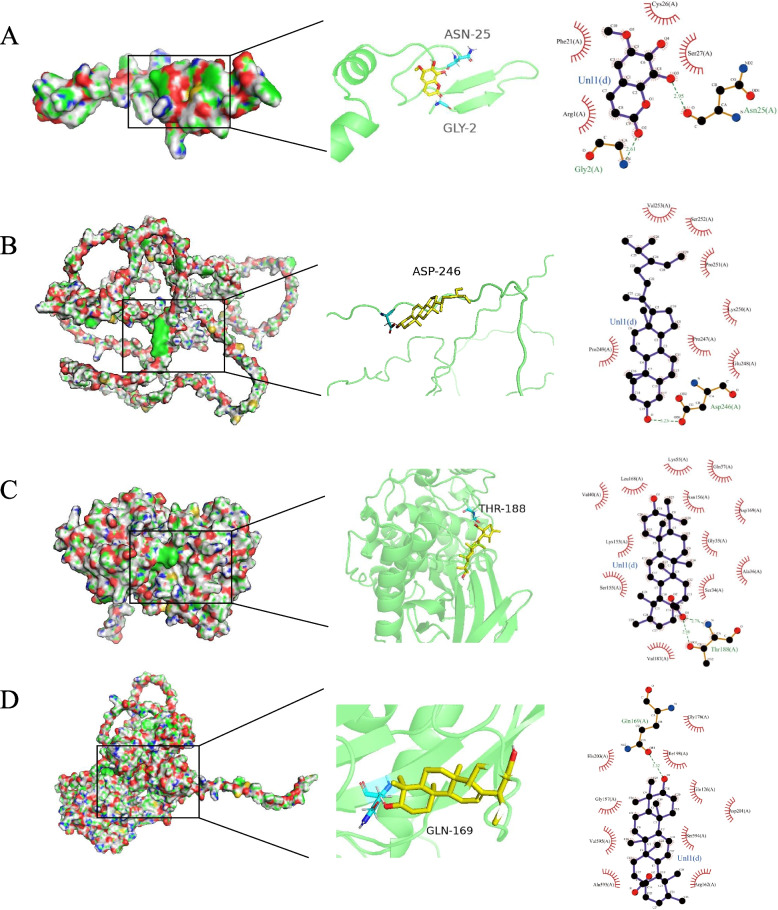
Conformations of molecular docking for (**A**) Fraxetin with PTGS2, **B** Beta-sitosterol with FOS, **C** Ursolic acid with MAPK8, and **D** Ursolic acid with MMP9

The molecular docking analyses were conducted between the four main compounds and the 22 target proteins. A binding energy less than − 5.0 kcal/mol indicates strong binding activity [29]. In present docking study, 35% (31/88) targets had a binding energy less than − 5.0 kcal/mol (Fig. 5). Ursolic acid binds to PTGS2 with a binding energy of − 7.98 kcal/mol, binds to IL1B at − 7.86 kcal/mol, and FOS at − 7.73 kcal/mol. Fraxetin binds to PTGS2 with a binding energy of − 5.31 kcal/mol. Beta-sitosterol binds to PTGS2 at − 5.45 kcal/mol, and CASP3 at − 6.39 kcal/mol (Table S7). The docking views showed that hydrophobic and hydrogen bond interactions are the primary modes of binding between compounds and proteins (Fig. 6A-D). Thus, we proposed that targeted regulation of these genes may be the mechanism of action of FC for UC treatment.

### The expression patterns of FC target genes in UC colonic tissues

**Fig. 7:**
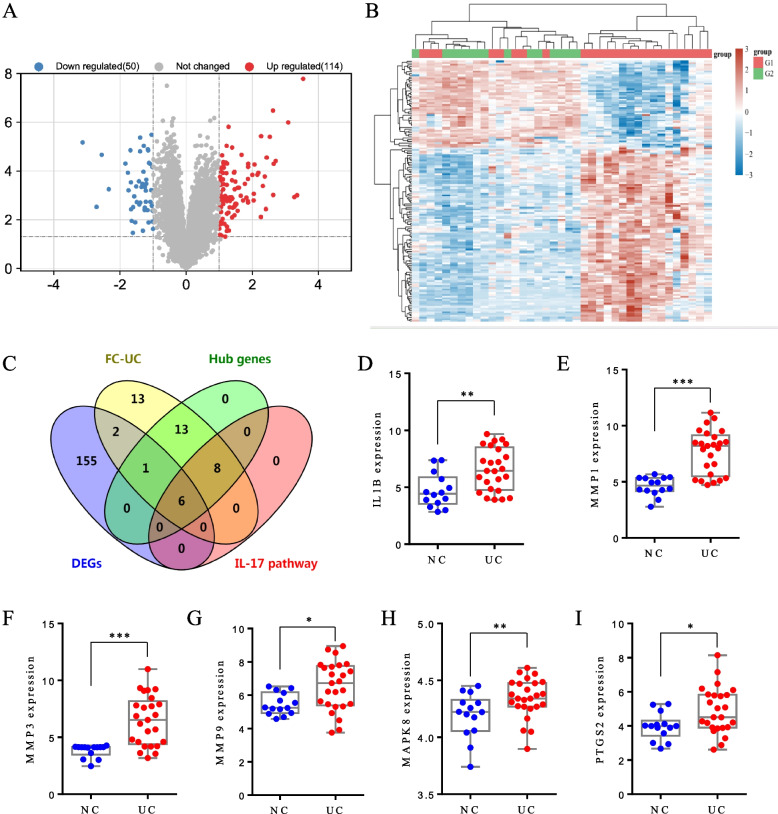
The expression profiles of FC-UC common targets in UC colonic tissues. **A** Volcano plot. **B** Heatmap of DEGs. **C** Venn diagram of targets shared by DEGs, FC-UC common genes, the hub genes and IL-17 signaling pathway-related genes. The expression distributions of (**D**) IL1B, (**E**) MMP1, (**F**) MMP3, (**G**) MMP9, (**H**) MAPK8, and (I) PTGS2 were showed as box plots, respectively. Data are presented as the mean ± SD. *P < 0.05, **P < 0.01, and ***P < 0.001 versus NC group

**Fig. 8:**
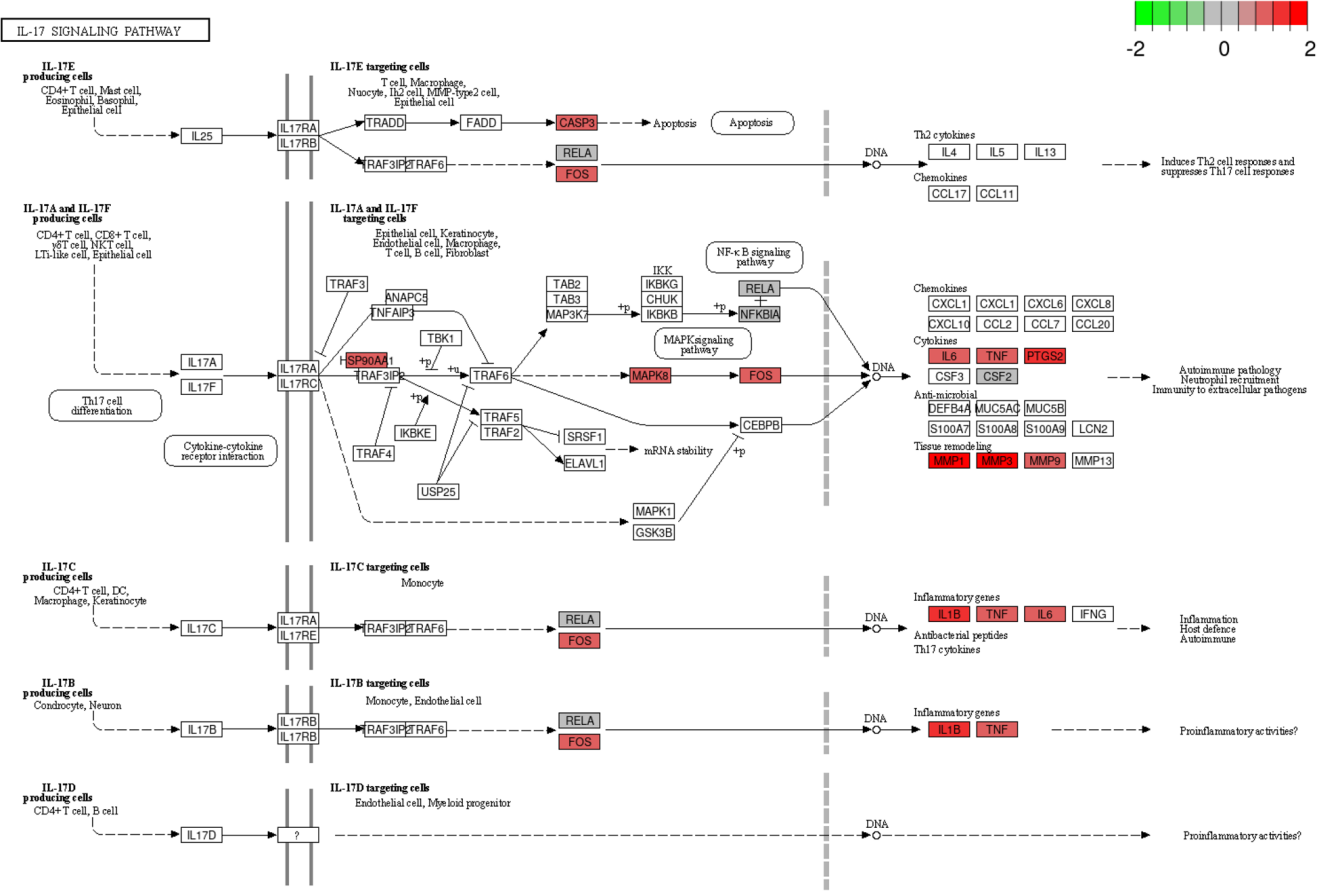
FC plays a role in UC treatment through IL-17 signaling pathway. FC potential targets are shown colored. Up-regulated genes are shown in red, down-regulated genes shown in green, and no changed genes shown in gray. Arrows and T-arrows represent the positive and negative effect, respectively. The mRNA expression data of FC targets were obtained from GEO datasets. Pathway analysis was performed using KEGG system [30] and under the permission of Kanehisa Laboratories

The mRNA expression data from two independent datasets (GSE22619 and GSE37283) were analyzed after normalization and batch removal (Fig. S3). A total of 164 differentially expressed genes (DEGs) were identified in UC colonic samples compared to the normal mucosal tissues, with 114 up-regulated and 50 down-regulated genes (Fig. 7A-B). Among of these DEGs, IL1B, PTGS2, MAPK8, MMP1, MMP3, and MMP9 were highly expressed in the colonic tissues of UC patients compared to that in the heathy controls (Fig. 7D-I). Notably, these six genes are the FC-UC common genes and enriched in IL-17 signaling pathway (Fig. 7C), which implied that these genes and IL-17 signaling pathway may be the targets of FC for UC treatment (Fig. 8, Table S8).

### FC attenuated DSS-induced colitis

**Fig. 9:**
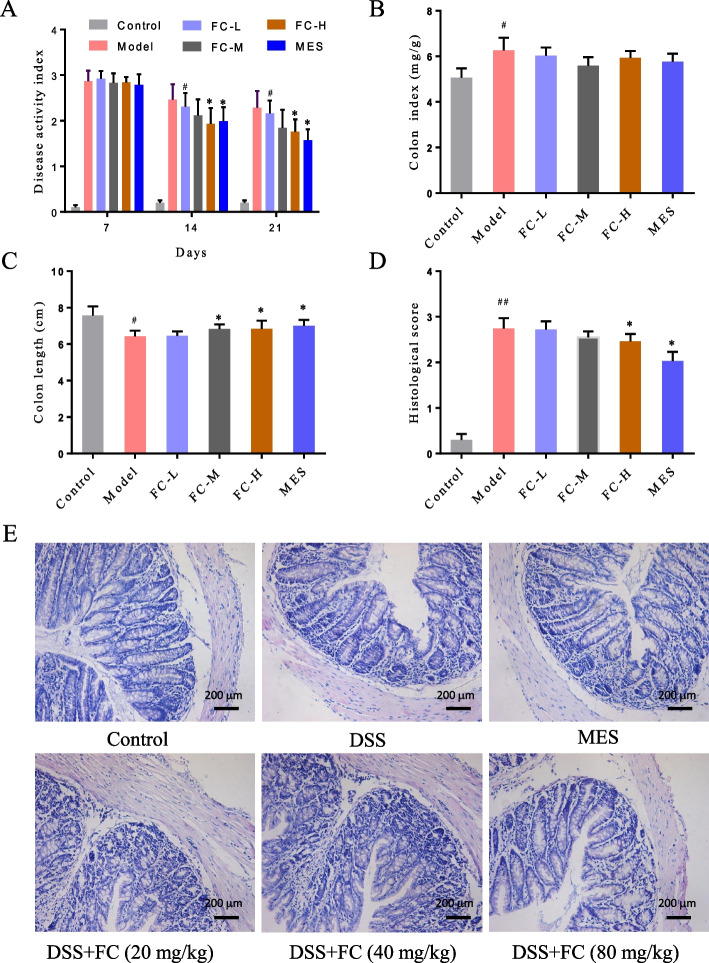
Therapeutic effects of FC on DSS-induced colitis. **A** Disease activity index. **B** Colon index. **C**. Colon length. **D** Histological score. **E** HE staining of colon tissues (200×, scale bar, 200 μm). Data are presented as the mean ± SD. ^*#*^*P* < 0.05 and ^*##*^*P* < 0.01 versus control group; ^*^P < 0.05 versus model group

To explore the therapeutic effect of FC on UC, a mouse colitis model was established and treated with FC. In comparison to the control group, mice induced with DSS had significant shortened colon, increased DAI and colon index, and higher histological score (Fig. 9A-D, S4). The colon tissues had obvious edema and epithelial destruction in the model group compared with that in the control group (Fig. 9E). However, these colitis symptoms and pathological changes were mitigated after FC treatment. The DAI, histological score, and colon length shortening were lower in FC-M and FC-H groups than that in the model group (Fig. 9A-D). All of these data suggested that FC ameliorated DSS-induce colitis.

### FC alleviated DSS-induced inflammation

**Fig. 10:**
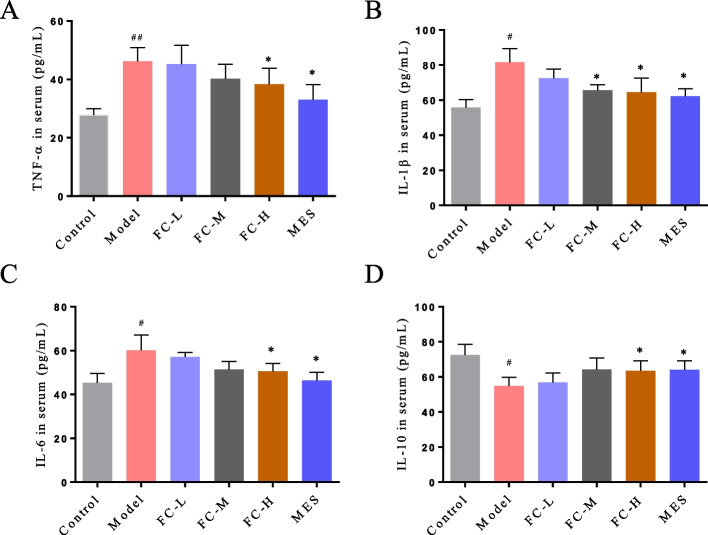
FC modulated inflammatory cytokine expression in DSS-induced colitis. The serum levels of (**A**) TNF-*α*, (**B**) IL-1β, (**C**) IL-6, and (**D**) IL-10 were evaluated by ELISA. Data are shown as the mean ± SD. ^*#*^*P* < 0.05 versus control group; ^*^P < 0.05 and^**^P < 0.05 versus model group

The serum contents of TNF-α, IL-1β and IL-6 were higher in the model group than that in the control group (Fig. 10A-C). While, the level of IL-10 was lower in the model group compared with that in the control group (Fig. 10D). After FC administration, the serum levels of TNF-α and IL-6 were decreased in FC-H group, and IL-1β was downregulated in FC-M and FC-H groups. Besides, IL-10 was upregulated in FC-H group compared with that in the model group (Fig. 10A-D). These results suggested that FC alleviated DSS-induced systemic inflammatory response in mice.

### FC modulated the expression of FC-UC common genes in colitis mice

**Fig. 11:**
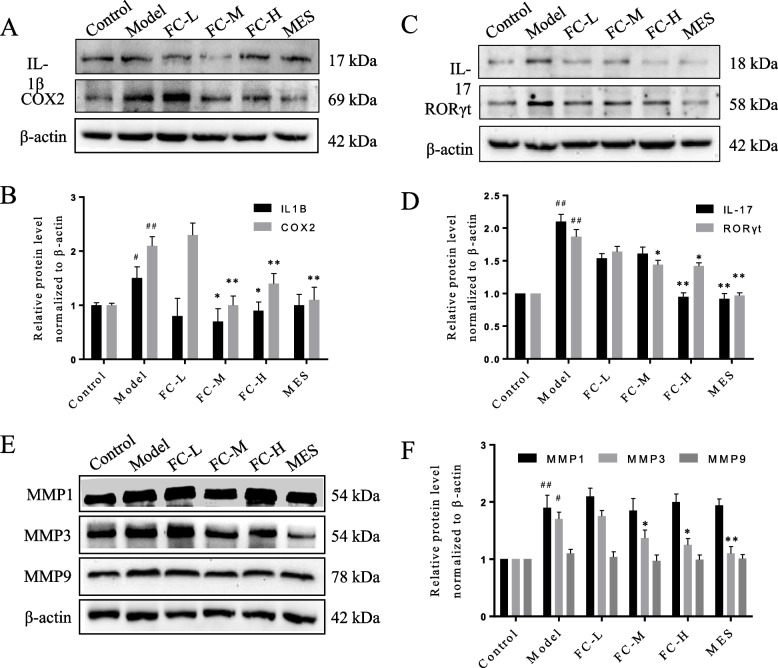
FC regulated the expression of key target genes. The protein expression levels of (**A**-**B**) IL-1β, COX2, (**C**-**D**) IL-17, RORγt, and (**E**-**F**) MMP1, MMP3 and MMP9 were measured by western blot. Data are shown as the mean ± SD, ^#^*P* < 0.05, ^##^*P* < 0.01 versus control group; *P < 0.05, **P < 0.01 versus model group

Several key targets including IL1β, COX2, IL-17, RORγt, MMP1 and MMP3 expression levels were validated by western blot. The protein levels of IL1β, PTGS2, IL-17 and RORγt were obvious decreased in FC-M and FC-H groups (Fig. 11A-D), and MMP3 was reduced in FC-H group (Fig. 11E-F), compared to that in the model group.

## Discussion

UC is a chronic, recurrent intestinal disease threatening the health of people worldwide [1]. Unfortunately, there is still no effective treatment to cure or prevent this disease. Traditional Chinese medicine (TCM) has been used in UC prevention and treatment over thousands of years [31]. Recently, it has drawn more attention to develop potential new UC therapeutics, attributing to its efficacy and low side effects [32, 33]. As one of the commonly used TCMs, FC is usually applied for treating diarrhea, bacillary dysentery, arthritis and cancer [34]. Identification of the pharmacological mechanisms of FC against UC will facilitate the development of novel therapies in UC treatment.

In this study, we used systems pharmacology to uncover the active compounds and potential targets of FC, and establish compound-target and target-disease relationships. Then, an in vivo animal experiment was used to validate the reliability of findings in the network pharmacology and molecular docking [35]. As the results achieved here, 16 active compounds and 28 hub target genes were identified, suggesting that FC exerted its anti-UC effects through multi-compounds and multi-targets. Ursolic acid, beta-sitosterol, fraxetin and esculetin were identified as the pivotal components with the highest degrees. In previous studies, ursolic acid has been proved to relive DSS-induced colitis in mice by reducing the content of malondialdehyde, IL-1β and TNF-α and increasing superoxide dismutase activity in mice colon tissues [36]. It also could attenuate experimental colitis by restoring intestinal flora homeostasis and regulating fatty acid metabolism [37]. In a *Drosophila* ulcerative colitis model, ursolic acid is demonstrated to restore the proliferation and differentiation of intestine stem cells and prevent intestine injury via inhibiting the JNK/JAK/STAT signaling pathway [38]. Beta-sitosterol is reported to alleviate microscopic appearances of DSS-induced colitis and increase the expression of antimicrobial peptides [39], and it could promote tissue repair by enhancing antioxidant defenses [40]. Fraxetin has been confirmed that it exhibits its intestinal anti-inflammatory activity through antioxidant property [41]. Esculetin has been demonstrated to ameliorate TNBS-induced rat colitis, and attenuate the expression of pro-inflammatory mediators TNF-α and IL-1β [42]. Overall, FC has a variety of active substances, and its anti-UC effect may be achieved by a variety of active ingredients acting on multiple targets, which is worthy of further study.

The PPI network analysis revealed that TP53, CASP3, IL6, PTGS2, TNF, IL1B, STAT3, MMP9 and NFKBIA were the core targets of FC. Ursolic acid was proved to exhibit anti-inflammation activities by acting on CASP3, ERK1 and JNK2 targets, inhibiting activation of inflammation-associated downstream factors ERK1, NF-κB and STAT3. It also downregulated the activities of IL-1β, IL-6, and TNF-α as well as expression of caspase-3 and caspase-9 [43]. Beta-sitosterol was demonstrated to reduce the expression levels of PTGS2 and NF-κB, and downregulate TNF-α, IL-1β and IL-6 [44]. Fraxetin was reported to mitigate the levels of IL-1β, IL-6, TNF-α and prostaglandin E2, improve the content of superoxide dismutase (SOD) and IL-10 in rats with enteritis [45]. Esculetin could attenuate iNOS and COX2 protein expression by inhibiting NF-κB pathway, and reduce LPS-induced elevated levels of IL-6, IL-1β and TNF-α in mice [46].

GO analysis revealed that FC was primarily associated with the biological processes of cytokine-mediated signaling pathway, positive regulation of apoptotic process, and cellular response to lipopolysaccharide. KEGG pathway enrichment analysis showed that FC exerted its therapeutic effects on UC by regulating IL-17 signaling pathway, TNF signaling pathway and Pathways in cancer. IL-17 is a proinflammatory factor in intestinal inflammation and is closely related to the pathogenesis of UC [47, 48]. IL-17A is the most important factor in the IL-17 family, which were highly expressed in biopsies of UC [49, 50]. IL-17A acts on immune and non-immune cells [51], actives NF-κB, MAPKs and C/EBP cascades [52–54], which induces the production of chemokines, inflammatory cytokines and acute phase reactive proteins, thus promotes the occurrence of inflammation. Inhibition of IL-17 signaling pathway by multi-targets and multi-channel inhibitors is an important approach for the treatment of UC [55].

In present study, IL-17 pathway was identified as the crucial pathway of FC in the UC treatment with the highest degree score via the compound-target-pathway network analysis. Molecular docking speculated that FC main components had high affinity with IL-17 signaling pathway related genes, and six of them (IL1B, MAPK8, PTGS2, MMP1, MMP3 and MMP9) were highly expressed in UC clinical samples validated by analysis of GEO expression datasets. Previous studies also demonstrated that THMs conquer UC via interfering IL-17 signaling pathway. For example, Qing Chang Suppository Powder improved DSS-induced colitis, and modulated the expression of mediators in IL-17 signaling pathway [56]. As the main components of FC, ursolic acid was previously reported to ameliorate the symptoms of autoimmune myasthenia gravis via inhibiting IL-17 and shifting Th17 to Th2 cytokines [57]. Ursolic acid also could alleviate autoimmune arthritis, decrease the levels of inflammatory cytokines, and reduce the number of Th17 cells [58]. Moreover, esculetin relived the lipopolysaccharide-induced acute lung injury, and inhibited the activation and/or expression of IL-17, AKT, ERK and RORγt [59]. In line with the results of network pharmacology approach, in vivo studies confirmed that FC alleviated DSS-induced colitis, reduced systemic inflammatory response, and diminished the expression of IL-17 signaling pathway mediators, IL-17A, RORγt, and tissue remodeling factors MMP1, MMP3 and MMP9.

Recently, network pharmacology has been widely applied to explore the action mechanism of THMs on various disease. This approach can be used to screen active compounds, describe the interaction between compounds and targets, and predict the molecular mechanism of THMs [29]. In addition to network pharmacology approach, we also used molecular docking and experimental studies to validate the predictive mechanism of FC for UC. However, there are some limitations in this research. For example, the predictive research relies on the various databases which maybe result in missing of important compounds and targets. The FC active compounds were retrieved from databases which are probably inconsistent with the ingredients in blood of UC patients. In addition, there are multiple targets and pathways were predicted in this study, but only IL-17 signaling pathway and related targets were validated in vivo studies. Therefore, the experimental studies for verification of the predicted molecular mechanisms of FC against UC are needed in future researches.

## Conclusions

In this research, ursolic acid, fraxetin, beta-sitosterol, and esculetin were identified as the main compounds, and MAPK8, IL6, RELA, TNF, IL1B, FOS, PTGS2, and MMP3 were considered as major targets of FC in UC treatment. Molecular docking verified that these compounds showed good binding interaction with these target proteins. FC exerted its therapeutic effect on UC primarily through IL-17 signaling pathway, TNF signaling pathway and Pathways in cancer. In vivo studies demonstrated that FC exerted its therapeutic effect on UC by inhibiting inflammatory cytokines release, and modulated IL-17 signaling pathway. Future studies are needed to determine the detailed molecular mechanism in mammals and human cell lines.

## Supplementary Information

**Additional file 1: Figure S1.** Therapeutic effects of FC on DSS-induced colitis. A. HE staining of colon tissues (200×, scale bar, 200 μm); B. Histological score. Data are presented as the mean ± SD (*n* = 10). ^*^P < 0.05 versus model group. **Figure S2.** Schematic graph of animal experiment. **Figure S3.** GEO datasets processing. (A) The boxplot of the normalized data. The x-axis represents samples and the y-axis represents the expression values. (B) PCA results before batch removal for multiple datasets. (C) PCA results after batch removal. **Figure S4.** Representative picture of the colon (*n* = 10).

**Additional file 2: Table S1.** Active compounds of Fraxini Cortex. **Table S2.** Predicted targets of the Fraxini Cortex compounds. **Table S3.** The hub genes enriched in biological processes. **Table S4.** The hub genes enriched in cellular components. **Table S5.** The hub genes enriched in molecular function. **Table S6.** KEGG pathway enrichment analysis of the hub genes. **Table S7.** Molecular docking of four compounds for UC targets. **Table S8.** The mRNA expression profile of FC targets in IL-17 signaling pathway.

**Additional file 3.**

## Data Availability

The datasets analysed in this study are available in the GEO repository, GSE22619 and GSE37283.

## Abbreviations

AKT: Protein kinase B
CASP3: Caspase-3
COX2: Cyclooxygenase-2
DAI: Disease activity index
DSS: Dextran Sulfate Sodium Salt
EGFR: Epidermal growth factor receptor
ERK: Extracellular signal-regulated kinase
FC: Fraxini Cortex
FOS: Protein c-Fos
GO: Gene ontology
HS: Histological score
IL1B: Interleukin-1 beta
IL6: Interleukin-6
IL-10: Interleukin-10
IL-17: Interleukin-17
iNOS: Inducible nitric oxide synthase
JAK: Janus kinase
JNK: c-Jun N-terminal kinase
KEGG: Kyoto Encyclopedia of Genes and Genomes
MAPK8: Mitogen-activated protein kinase 8
MES: Mesalazine
NFKBIA: NF-kappa-B inhibitor alpha
NOD: Nucleotide oligomerization domain
PI3K: Phosphoinositide-3-kinase
PPI: Protein-protein interaction
PTGS2: Prostaglandin G/H synthase 2
PVDF: Polyvinylidene fluoride
RELA: Transcription factor p65
RORγt: Retinoic acid-related orphan receptor γt
SDS-PAGE: Sodium dodecyl sulfate polyacrylamide gel electrophoresis
STAT3: Signal transducer and activator of transcription 3
TCM: Traditional Chinese Medicine
TCMSP: Traditional Chinese Medicine Systems Pharmacology Database
TGFB1: Transforming growth factor beta-1
Th17: T helper type 17
TLR: Toll-like receptors
TNF: Tumor necrosis factor
UC: Ulcerative colitis

## References

[1] Ungaro R, Mehandru S, Allen PB, Peyrin-Biroulet L, Colombel JF (2017). Ulcerative colitis. Lancet..

[2] Ng SC, Shi HY, Hamidi N, Underwood FE, Tang W, Benchimol EI (2017). Worldwide incidence and prevalence of inflammatory bowel disease in the 21st century: a systematic review of population-based studies. Lancet..

[3] Du L, Ha C (2020). Epidemiology and pathogenesis of ulcerative colitis. Gastroenterol Clin N Am.

[4] Porter RJ, Kalla R, Ho GT. Ulcerative colitis: recent advances in the understanding of disease pathogenesis. F1000Research. 2020;9:F1000 Faculty Rev-294. 10.12688/f1000research.20805.1 PMC7194476 32399194

[5] Hirten RP, Sands BE (2021). New therapeutics for ulcerative colitis. Annu Rev Med.

[6] Kucharzik T, Koletzko S, Kannengiesser K, Dignass A (2020). Ulcerative colitis-diagnostic and therapeutic algorithms. Deutsches Arzteblatt Int.

[7] Liang C, Ju W, Pei S, Tang Y, Xiao Y. Pharmacological activities and synthesis of Esculetin and its derivatives: a Mini-review. Molecules. 2017;22(3):387. 10.3390/molecules22030387 PMC6155195 28257115

[8] Zhao CN, Yao ZL, Yang D, Ke J, Wu QL, Li JK, et al. Chemical constituents from Fraxinus hupehensis and their antifungal and herbicidal activities. Biomolecules. 2020;10(1):74. 10.3390/biom10010074 PMC7022268 31906487

[9] Li W, Li W, Yu J, Liu F, Zang L, Xiao X (2020). Fraxin inhibits lipopolysaccharide-induced inflammatory cytokines and protects against endotoxic shock in mice. Fundam Clin Pharmacol.

[10] Li WF, Li WQ, Zang LL, Liu F, Yao Q, Zhao JM (2019). Fraxin ameliorates lipopolysaccharide-induced acute lung injury in mice by inhibiting the NF-KB and NLRP3 signalling pathways. Int Immunopharmacol.

[11] Li JJ, Zhou SY, Zhang H, Lam KH, Lee SM, Yu PH (2015). Cortex Fraxini (Qingpi) protects rat Pheochromocytoma cells against 6-Hydroxydopamine-induced apoptosis. Parkinsons Dis.

[12] Wu B, Wang R, Li SN, Wang YY, Song FX, Gu YQ (2019). Antifibrotic effects of Fraxetin on carbon tetrachloride-induced liver fibrosis by targeting NF-kappa B/IkB alpha, MAPKs and Bcl-2/Bax pathways. Pharmacol Rep.

[13] Yang L, Meng X, Yu X, Kuang H (2017). Simultaneous determination of anemoside B4, phellodendrine, berberine, palmatine, obakunone, esculin, esculetin in rat plasma by UPLC-ESI-MS/MS and its application to a comparative pharmacokinetic study in normal and ulcerative colitis rats. J Pharm Biomed Anal.

[14] Tsai JC, Tsai S, Chang WC (2004). Effect of ethanol extracts of three Chinese medicinal plants with anti-diarrheal properties on ion transport of the rat intestinal epithelia. J Pharmacol Sci.

[15] Wu XF, Ouyang ZJ, Feng LL, Chen G, Guo WJ, Shen Y (2014). Suppression of NF-kappaB signaling and NLRP3 inflammasome activation in macrophages is responsible for the amelioration of experimental murine colitis by the natural compound fraxinellone. Toxicol Appl Pharmacol.

[16] Tian X, Peng Z, Luo S, Zhang S, Li B, Zhou C (2019). Aesculin protects against DSS-induced colitis though activating PPARgamma and inhibiting NF-small ka, CyrillicB pathway. Eur J Pharmacol.

[17] Wang SK, Chen TX, Wang W, Xu LL, Zhang YQ, Jin Z (2022). Aesculetin exhibited anti-inflammatory activities through inhibiting NF-small ka, CyrillicB and MAPKs pathway in vitro and in vivo. J Ethnopharmacol.

[18] Han Z, Tan X, Sun J, Wang T, Yan G, Wang C (2021). Systems pharmacology and transcriptomics reveal the mechanisms of Sanhuang decoction enema in the treatment of ulcerative colitis with additional Candida albicans infection. Chin Med.

[19] Wang ZY, Wang X, Zhang DY, Hu YJ, Li S (2022). Traditional Chinese medicine network pharmacology: development in new era under guidance of network pharmacology evaluation method guidance. Zhongguo Zhong Yao Za Zhi.

[20] Li SCY, Ding QY, Dai JY, Duan XC, Hu YJ (2021). Network pharmacology evaluation method guidance-draft. World J Tradit Chin Med.

[21] Lepage P, Hasler R, Spehlmann ME, Rehman A, Zvirbliene A, Begun A (2011). Twin study indicates loss of interaction between microbiota and mucosa of patients with ulcerative colitis. Gastroenterology..

[22] Pekow J, Dougherty U, Huang Y, Gometz E, Nathanson J, Cohen G (2013). Gene signature distinguishes patients with chronic ulcerative colitis harboring remote neoplastic lesions. Inflamm Bowel Dis.

[23] Zhou Y, Zhang X, Li C, Yuan X, Han L, Li Z (2018). Research on the pharmacodynamics and mechanism of Fraxini cortex on hyperuricemia based on the regulation of URAT1 and GLUT9. Biomed Pharmacother.

[24] Chassaing B, Aitken JD, Malleshappa M, Vijay-Kumar M (2014). Dextran sulfate sodium (DSS)-induced colitis in mice. Curr Protoc Immunol.

[25] Kim JJ, Shajib MS, Manocha MM, Khan WI. Investigating intestinal inflammation in DSS-induced model of IBD. J Vis Exp. 2012;(60):3678. 10.3791/3678 PMC3369627 22331082

[26] Ma K, Chen M, Liu J, Ge Y, Wang T, Wu D (2021). Sodium houttuyfonate attenuates dextran sulfate sodium associated colitis precolonized with Candida albicans through inducing beta-glucan exposure. J Leukoc Biol.

[27] Xie SZ, Liu B, Ye HY, Li QM, Pan LH, Zha XQ (2019). Dendrobium huoshanense polysaccharide regionally regulates intestinal mucosal barrier function and intestinal microbiota in mice. Carbohydr Polym.

[28] Tang X, Li X, Wang Y, Zhang Z, Deng A, Wang W (2019). Butyric acid increases the therapeutic effect of EHLJ7 on ulcerative colitis by inhibiting JAK2/STAT3/SOCS1 signaling pathway. Front Pharmacol.

[29] Chen ZQ, Lin T, Liao XZ, Li ZY, Lin RT, Qi XJ, et al. Network pharmacology based research into the effect and mechanism of Yinchenhao decoction against cholangiocarcinoma. Chin Med. 2021;16(1):13. 10.1186/s13020-021-00423-4 PMC7818939 33478536

[30] Kanehisa M, Furumichi M, Sato Y, Kawashima M, Ishiguro-Watanabe M (2023). KEGG for taxonomy-based analysis of pathways and genomes. Nucleic Acids Res.

[31] Sun YX, Wang X, Liao X, Guo J, Hou WB, Wang X (2021). An evidence mapping of systematic reviews and meta-analysis on traditional Chinese medicine for ulcerative colitis. BMC Complement Med Ther.

[32] Liu Y, Li BG, Su YH, Zhao RX, Song P, Li H (2022). Potential activity of traditional Chinese medicine against ulcerative colitis: a review. J Ethnopharmacol.

[33] Zhang X, Zhang L, Chan JCP, Wang X, Zhao C, Xu Y (2022). Chinese herbal medicines in the treatment of ulcerative colitis: a review. Chin Med.

[34] Guo S, Guo T, Cheng N, Liu Q, Zhang Y, Bai L (2017). Hepatoprotective standardized EtOH-water extract from the seeds of Fraxinus rhynchophylla Hance. J Tradit Complement Med.

[35] Shang T, Yu Q, Ren T, Wang XT, Zhu H, Gao JM (2019). Xuebijing injection maintains GRP78 expression to prevent Candida albicans-induced epithelial death in the kidney. Front Pharmacol.

[36] Liu B, Piao X, Guo L, Liu S, Chai F, Gao L (2016). Ursolic acid protects against ulcerative colitis via anti-inflammatory and antioxidant effects in mice. Mol Med Rep.

[37] Sheng Q, Li F, Chen G, Li J, Li J, Wang Y (2021). Ursolic acid regulates intestinal microbiota and inflammatory cell infiltration to prevent ulcerative colitis. J Immunol Res.

[38] Wei T, Wu L, Ji X, Gao Y, Xiao G. Ursolic acid protects sodium dodecyl sulfate-induced Drosophila ulcerative colitis model by inhibiting the JNK signaling. Antioxidants. 2022;11(2):426. 10.3390/antiox11020426 PMC8869732 35204308

[39] Ding K, Tan YY, Ding Y, Fang Y, Yang X, Fang J (2019). beta-Sitosterol improves experimental colitis in mice with a target against pathogenic bacteria. J Cell Biochem.

[40] Abbas MM, Al-Rawi N, Abbas MA, Al-Khateeb I (2019). Naringenin potentiated beta-sitosterol healing effect on the scratch wound assay. Res Pharm Sci.

[41] Witaicenis A, Seito LN, da Silveira CA, de Almeida LD, Luchini AC, Rodrigues-Orsi P (2014). Antioxidant and intestinal anti-inflammatory effects of plant-derived coumarin derivatives. Phytomedicine.

[42] Yum S, Jeong S, Lee S, Kim W, Nam J, Jung Y (2015). HIF-prolyl hydroxylase is a potential molecular target for esculetin-mediated anti-colitic effects. Fitoterapia..

[43] Ma X, Zhang Y, Wang Z, Shen Y, Zhang M, Nie Q, et al. Ursolic acid, a natural nutraceutical agent, targets Caspase3 and alleviates inflammation-associated downstream signal transduction. Mol Nutr Food Res. 2017;61(12):1700332. 10.1002/mnfr.201700332 PMC5765441 28801966

[44] Zhang F, Liu Z, He X, Li Z, Shi B, Cai F (2020). beta-Sitosterol-loaded solid lipid nanoparticles ameliorate complete Freund's adjuvant-induced arthritis in rats: involvement of NF-small ka, CyrillicB and HO-1/Nrf-2 pathway. Drug Deliv.

[45] Miao Z, Zhang L, Gu M, Huang J, Wang X, Yan J (2021). Preparation of Fraxetin long circulating liposome and its anti-enteritis effect. AAPS PharmSciTech.

[46] Zhu L, Nang C, Luo F, Pan H, Zhang K, Liu J (2016). Esculetin attenuates lipopolysaccharide (LPS)-induced neuroinflammatory processes and depressive-like behavior in mice. Physiol Behav.

[47] Zhou C, Wu D, Jawale C, Li Y, Biswas PS, McGeachy MJ (2021). Divergent functions of IL-17-family cytokines in DSS colitis: insights from a naturally-occurring human mutation in IL-17F. Cytokine..

[48] Fauny M, Moulin D, D'Amico F, Netter P, Petitpain N, Arnone D (2020). Paradoxical gastrointestinal effects of interleukin-17 blockers. Ann Rheum Dis.

[49] Aghamohamadi E, Asri N, Odak A, Rostami-Nejad M, Chaleshi V, Hajinabi Y, et al. Gene expression analysis of intestinal IL-8, IL-17 a and IL-10 in patients with celiac and inflammatory bowel diseases. Mol Biol Rep. 2022;49(7):6085-91. 10.1007/s11033-022-07397-y 35526253

[50] Lucaciu LA, Ilies M, Vesa SC, Seicean R, Din S, Iuga CA, et al. Serum interleukin (IL)-23 and IL-17 profile in inflammatory bowel disease (IBD) patients could differentiate between severe and non-severe disease. J Pers Med. 2021;11(11):1130. 10.3390/jpm11111130 PMC8621192 34834482

[51] Grivennikov SI, Wang K, Mucida D, Stewart CA, Schnabl B, Jauch D (2012). Adenoma-linked barrier defects and microbial products drive IL-23/IL-17-mediated tumour growth. Nature..

[52] Akitsu A, Iwakura Y (2018). Interleukin-17-producing gammadelta T (gammadelta17) cells in inflammatory diseases. Immunology..

[53] Song X, Qian Y (2013). The activation and regulation of IL-17 receptor mediated signaling. Cytokine..

[54] Wei L, Liu M, Xiong H, Peng B (2018). Up-regulation of IL-23 expression in human dental pulp fibroblasts by IL-17 via activation of the NF-kappaB and MAPK pathways. Int Endod J.

[55] Fitzpatrick LR, Stonesifer E, Small JS, Liby KT (2014). The synthetic triterpenoid (CDDO-Im) inhibits STAT3, as well as IL-17, and improves DSS-induced colitis in mice. Inflammopharmacology..

[56] Zhou G, Kong WS, Li ZC, Xie RF, Yu TY, Zhou X (2021). Effects of Qing Chang suppository powder and its ingredients on IL-17 signal pathway in HT-29 cells and DSS-induced mice. Phytomedicine.

[57] Xu H, Zhang M, Li XL, Li H, Yue LT, Zhang XX (2015). Low and high doses of ursolic acid ameliorate experimental autoimmune myasthenia gravis through different pathways. J Neuroimmunol.

[58] Baek SY, Lee J, Lee DG, Park MK, Lee J, Kwok SK (2014). Ursolic acid ameliorates autoimmune arthritis via suppression of Th17 and B cell differentiation. Acta Pharmacol Sin.

[59] Lee HC, Liu FC, Tsai CN, Chou AH, Liao CC, Yu HP (2020). Esculetin ameliorates lipopolysaccharide-induced acute lung injury in mice via modulation of the AKT/ERK/NF-kappaB and RORgammat/IL-17 pathways. Inflammation..

