# Supplementary material for: Deciphering the pharmacological mechanisms of Fraxini Cortex for ulcerative colitis treatment based on network pharmacology and in vivo studies
Source: BMC Complement Med Ther. 2023 May 9;23:152. doi: 10.1186/s12906-023-03983-0 (PMC10170718; doi:10.1186/s12906-023-03983-0)
Supplement: Supplementary file 1 — Additional file 1: Figure S1. Therapeutic effects of FC on DSS-induced colitis. A. HE staining of colon tissues (200×, scale bar, 200 μm); B. Histological score. Data are presented as the mean ± SD (n = 10). *P < 0.05 versus model group. Figure S2. Schematic graph of animal experiment. Figure S3. GEO datasets processing. (A) The boxplot of the normalized data. The x-axis represents samples and the y-axis represents the expression values. (B) PCA results before batch removal for multiple datasets. (C) PCA results after batch removal. Figure S4. Representative picture of the colon (n = 10). [file 12906_2023_3983_MOESM1_ESM.docx]

Supplementary figure 1


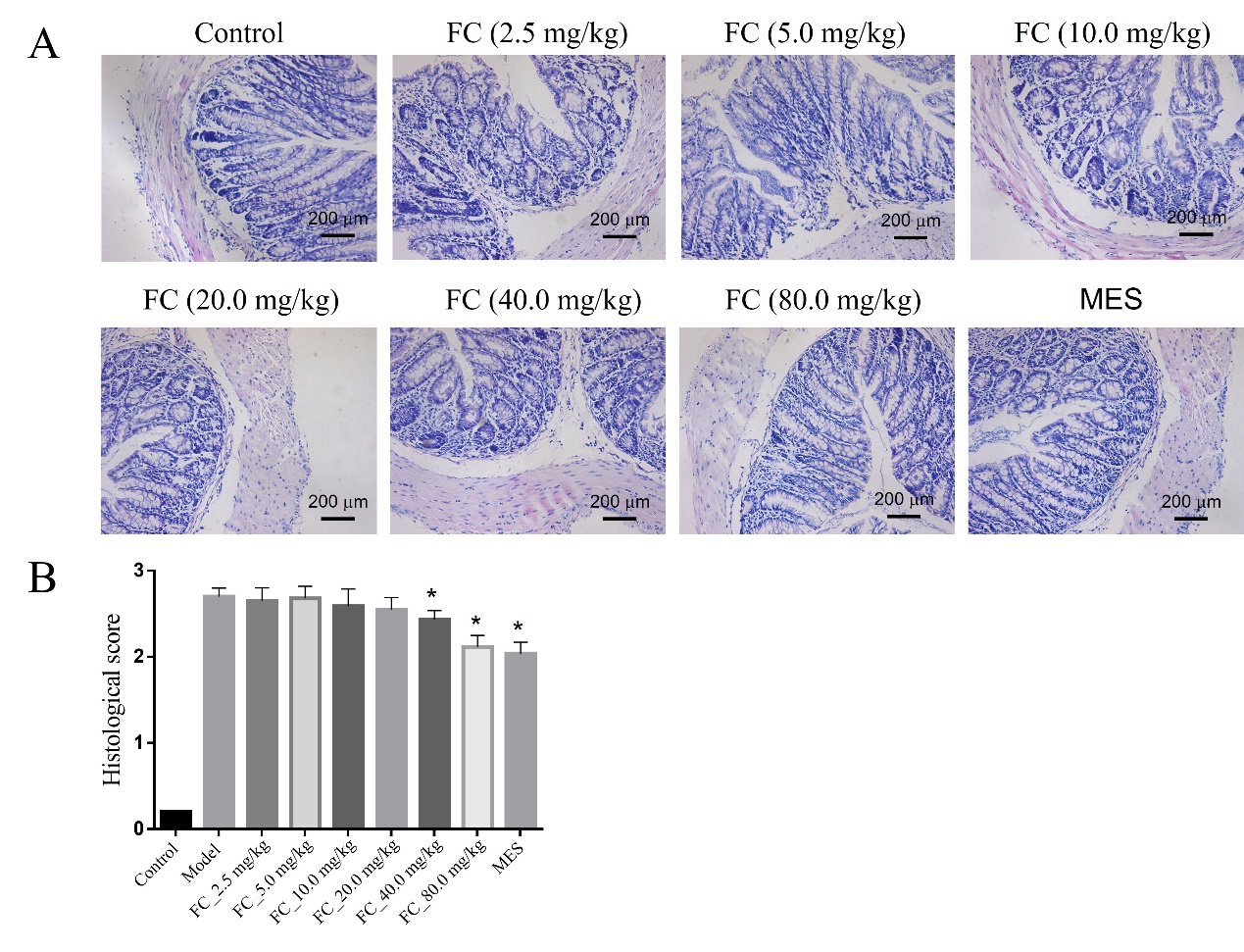


Figure S1 Therapeutic effects of FC on DSS-induced colitis

A. HE staining of colon tissues (200×, scale bar, 200μm); B. Histological score. Data are presented as the mean ± SD (n=10). ^*^*P*< 0.05 versus model group.

Supplementary figure 2


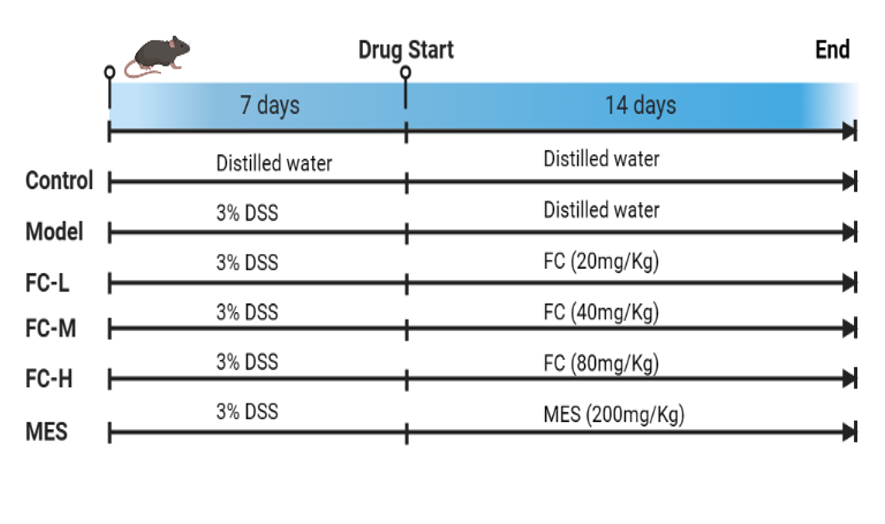


Figure S2 Schematic graph of animal experiment

Supplementary figure 3


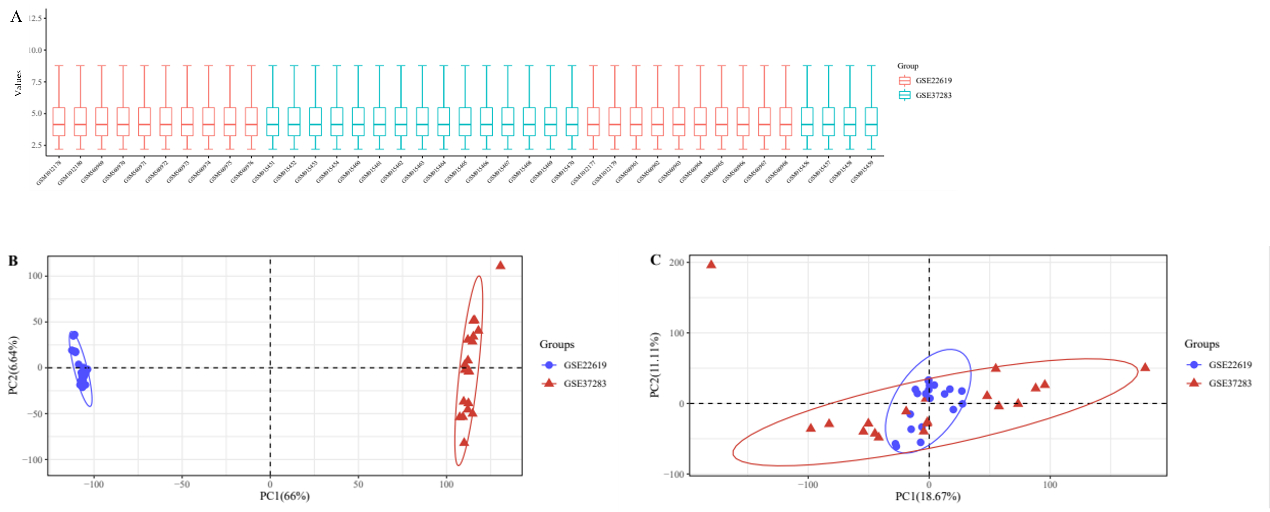


Figure S3 GEO datasets processing. (A) The boxplot of the normalized data. The x-axis represent samples and the y-axis represent the expression values. (B) PCA results before batch removal for multiple datasets. (C) PCA results after batch removal.

Supplementary figure 4


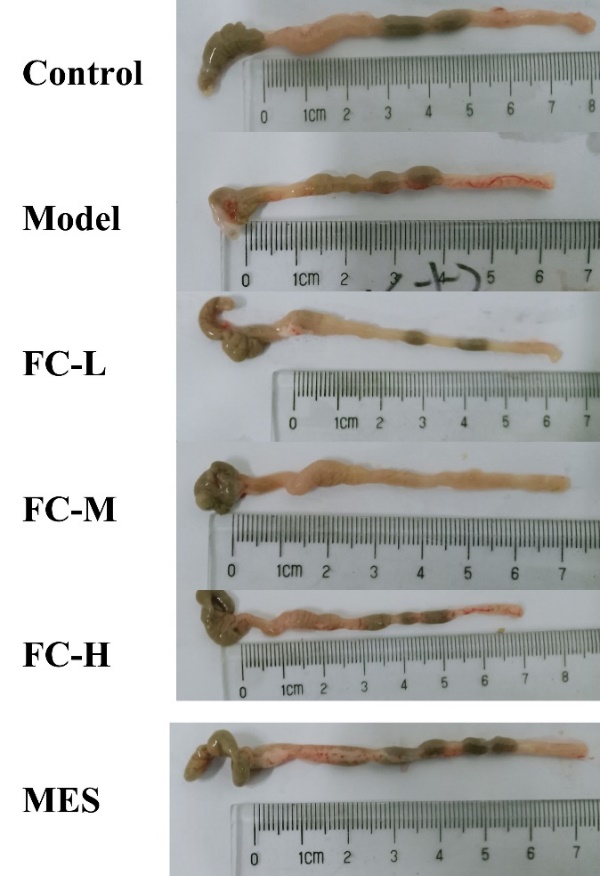


Figure S4 Representative picture of the colon (n=10).
