# Supplementary material for: Deciphering the pharmacological mechanisms of Fraxini Cortex for ulcerative colitis treatment based on network pharmacology and in vivo studies
Source: BMC Complement Med Ther. 2023 May 9;23:152. doi: 10.1186/s12906-023-03983-0 (PMC10170718; doi:10.1186/s12906-023-03983-0)

**Figure 11A**

IL-1β (17 kDa)


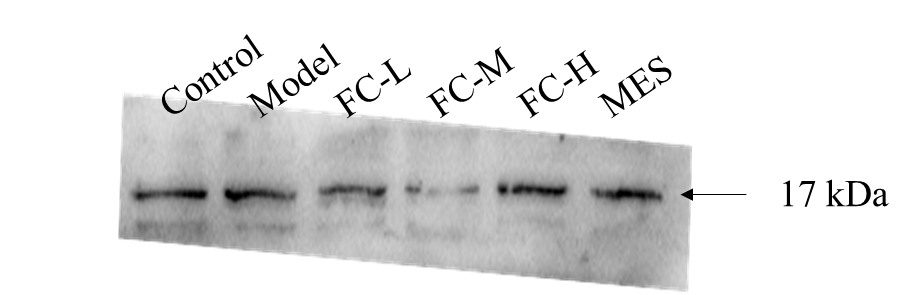


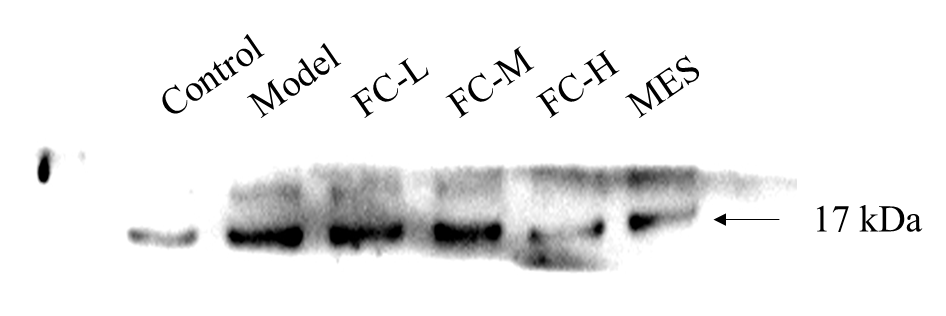


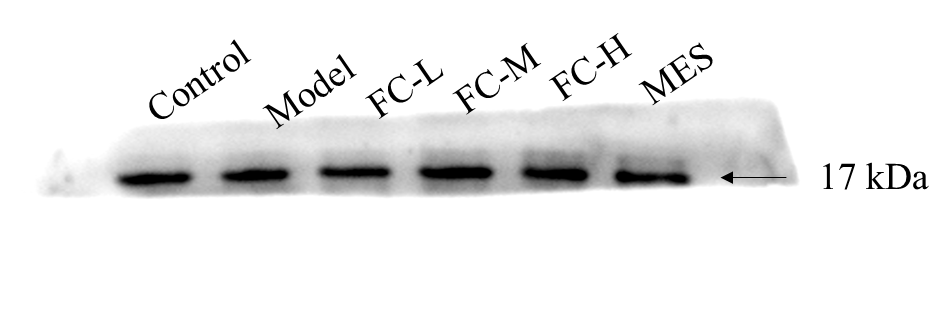


**COX2 (69 kDa)**


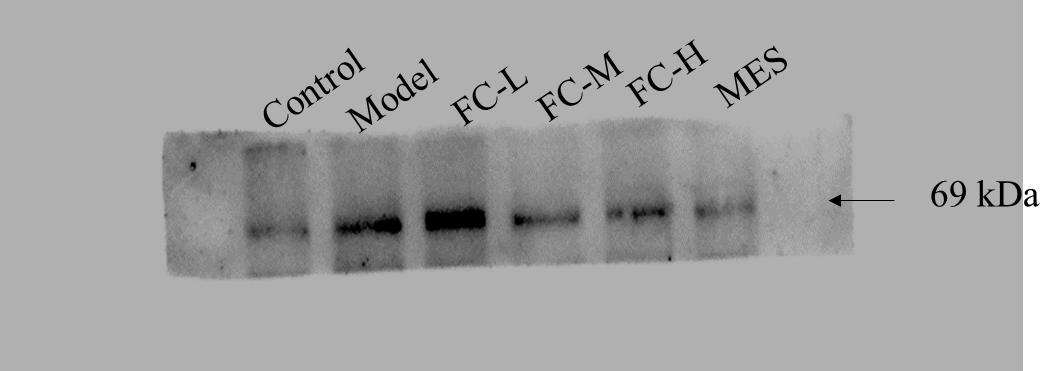


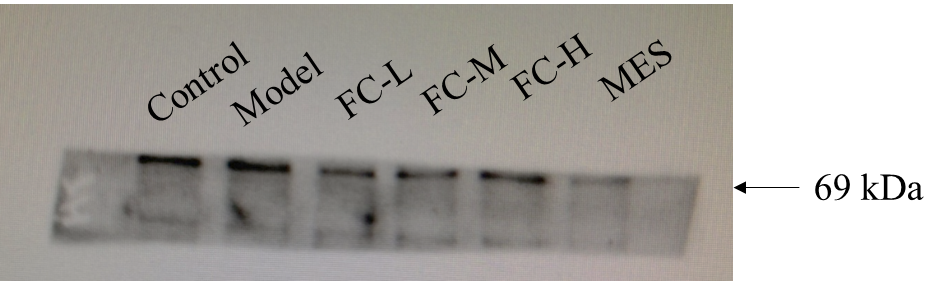


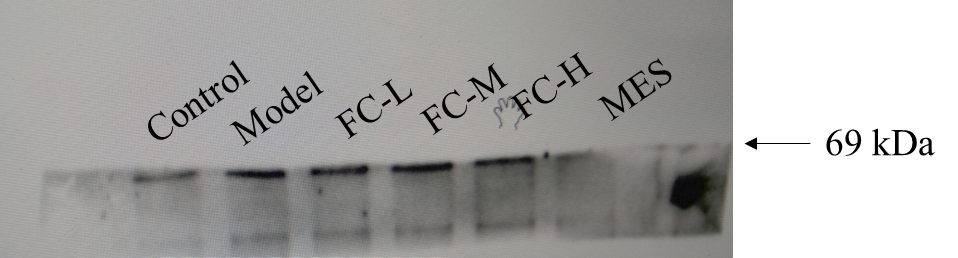


**β-actin (42 kDa)**


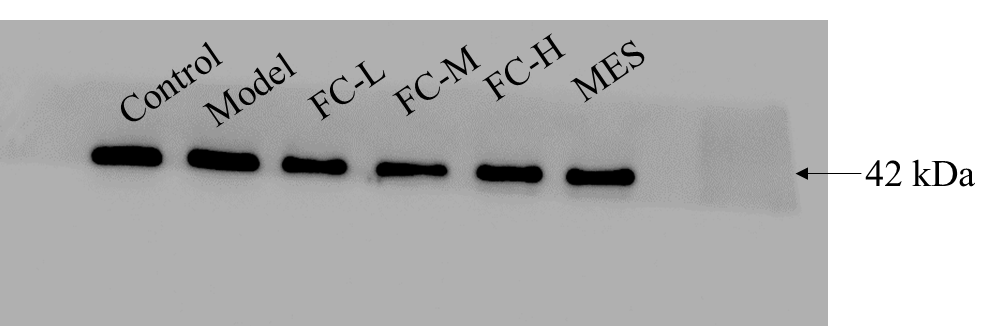


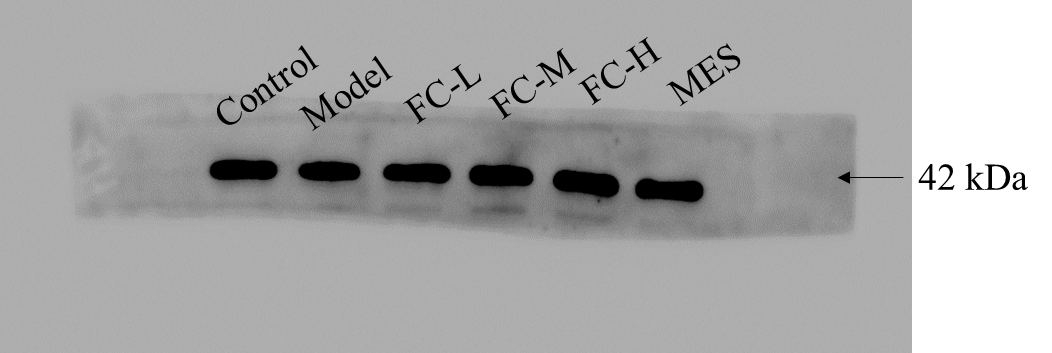


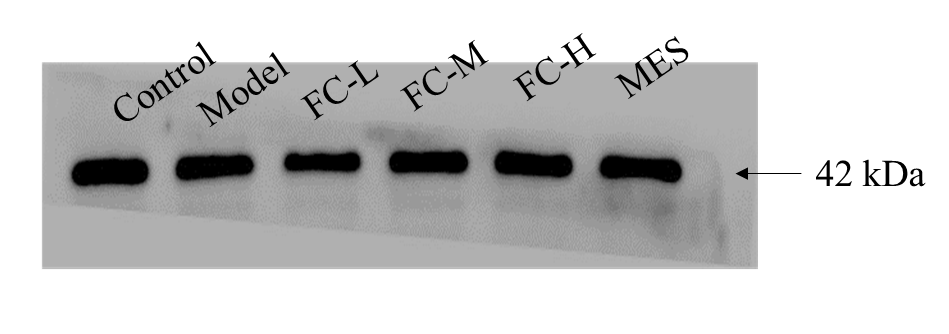


**Figure 11C**

**1L-17 (18 kDa)**


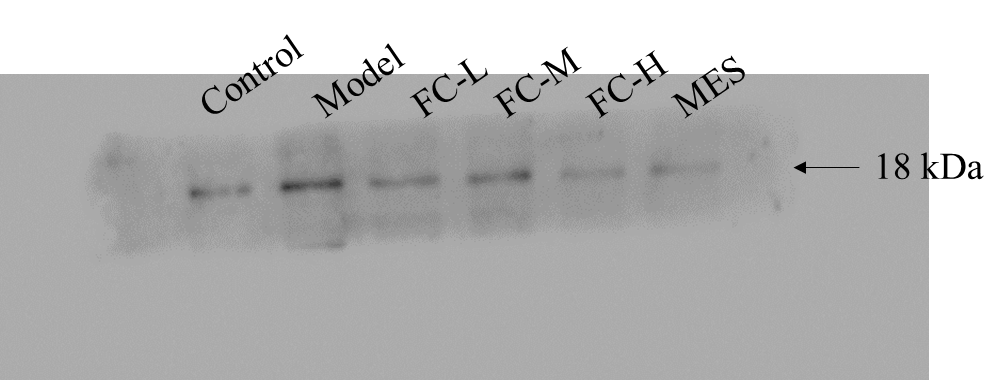


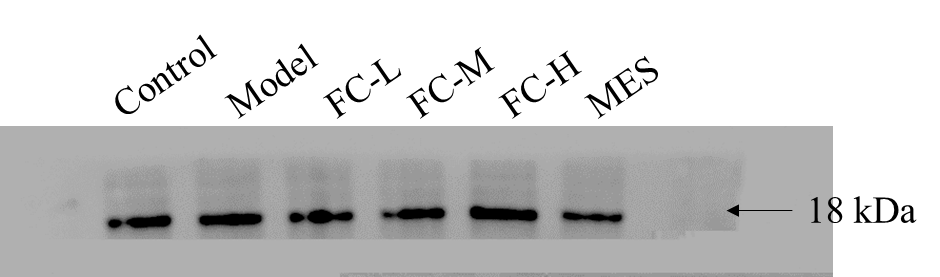


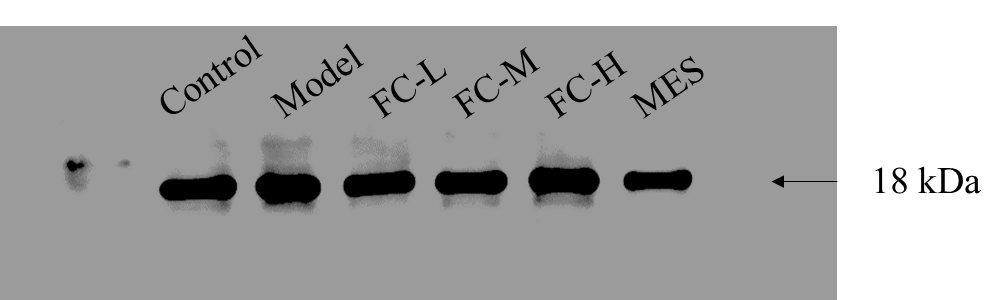


**RORγt (58 kDa)**


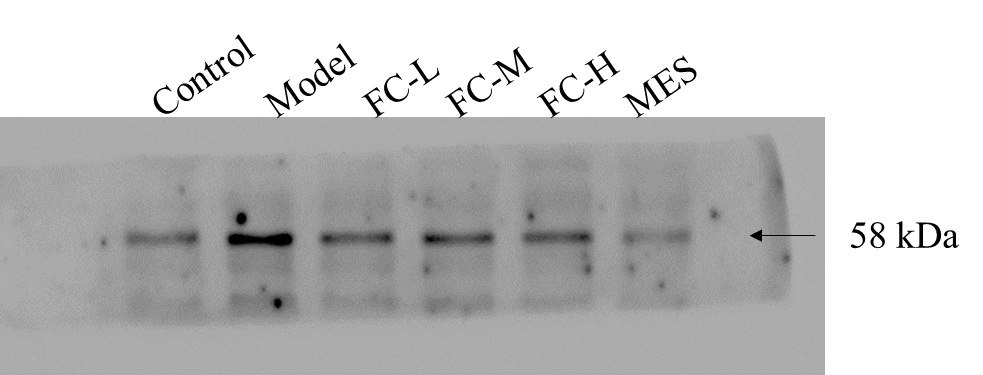


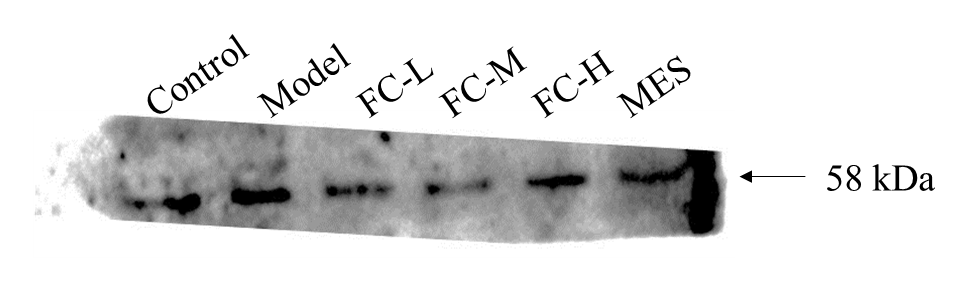


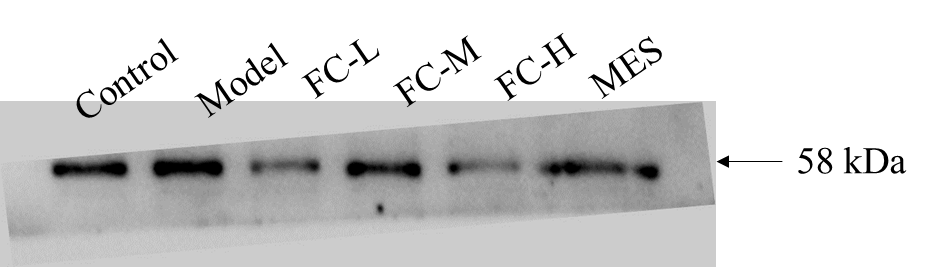


**β-actin (42 kDa)**

**
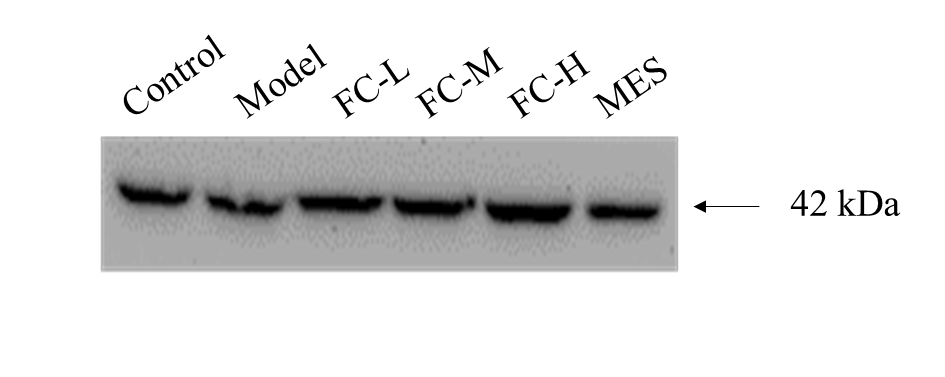
**


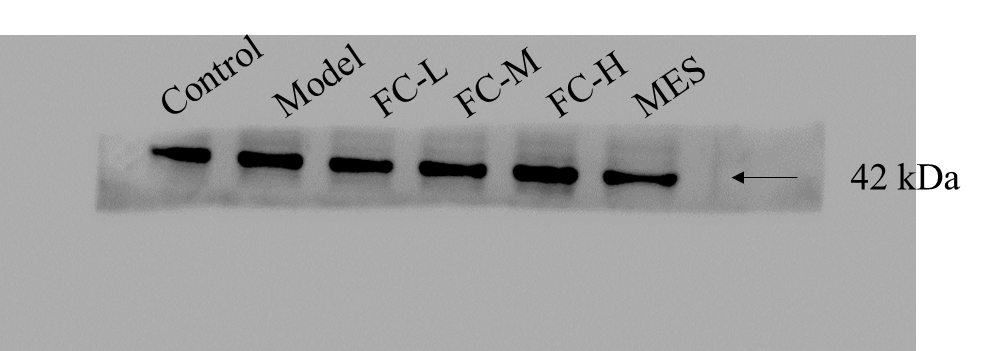


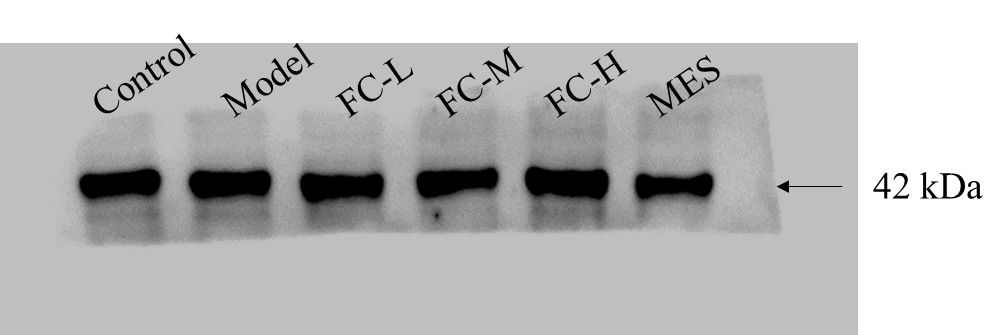


**Figure 11C**

**MMP1 (54 kDa)**


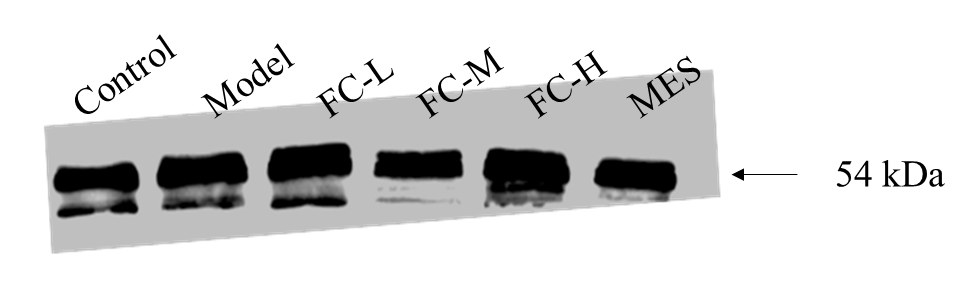


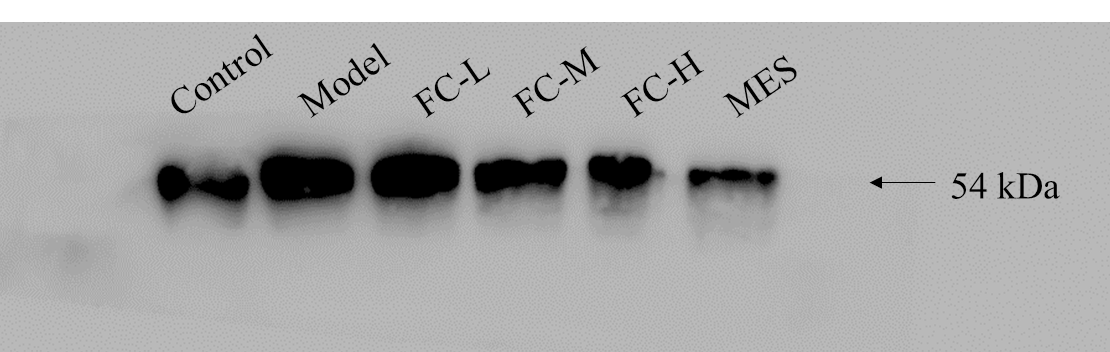


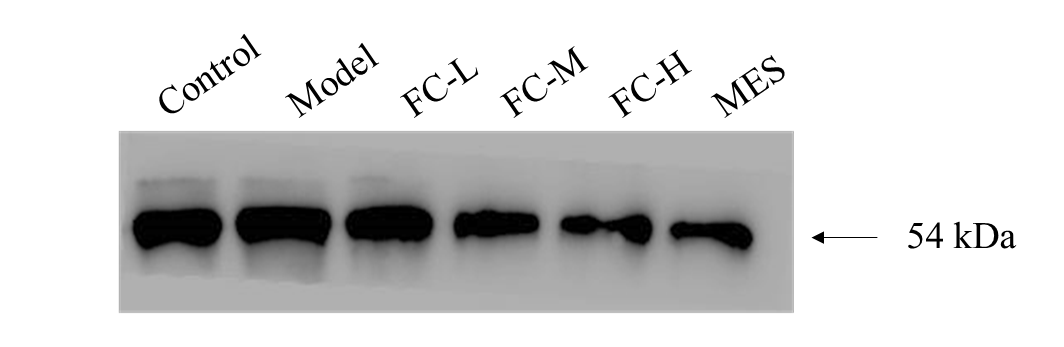


**MMP3 (54 kDa)**


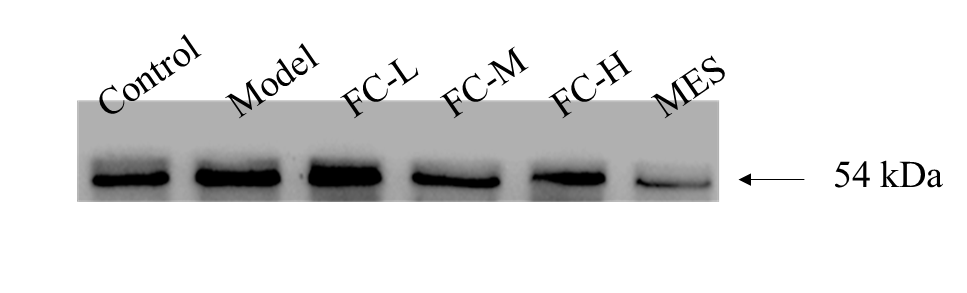


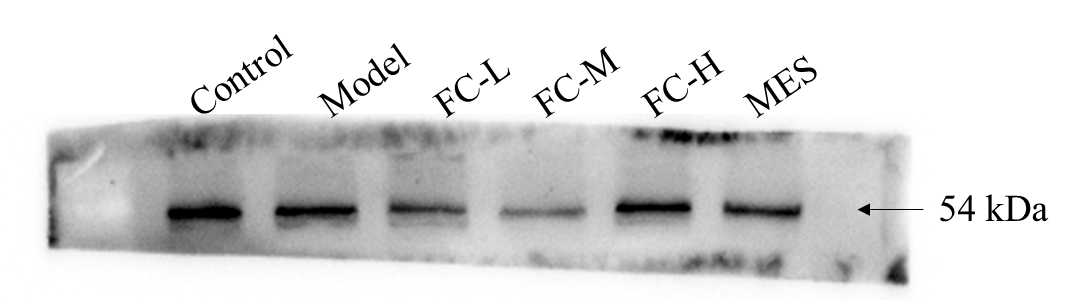


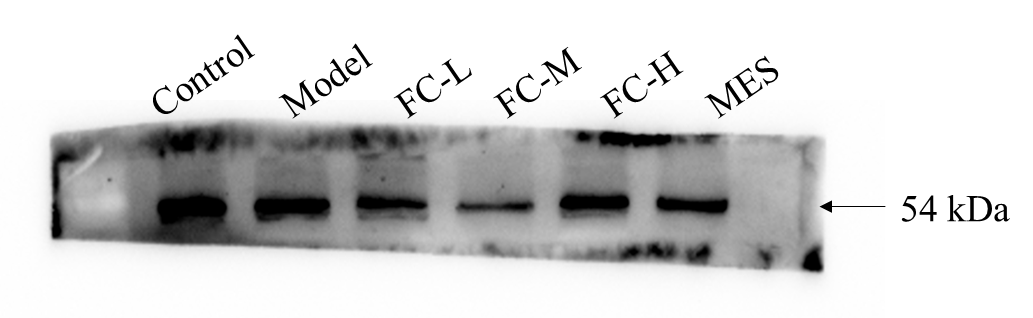


**MMP9 (78 kDa)**

**
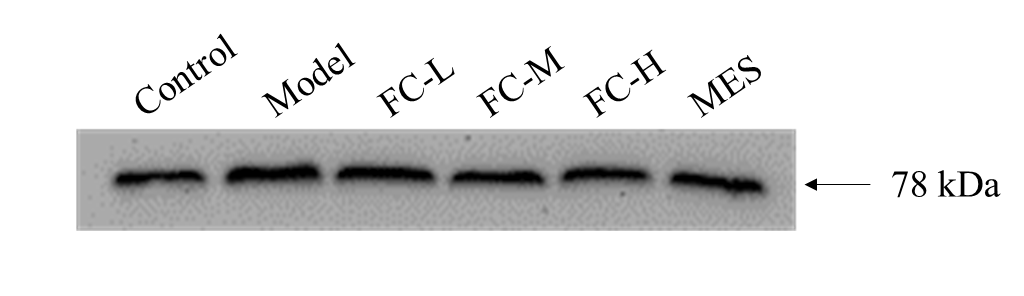
**

**
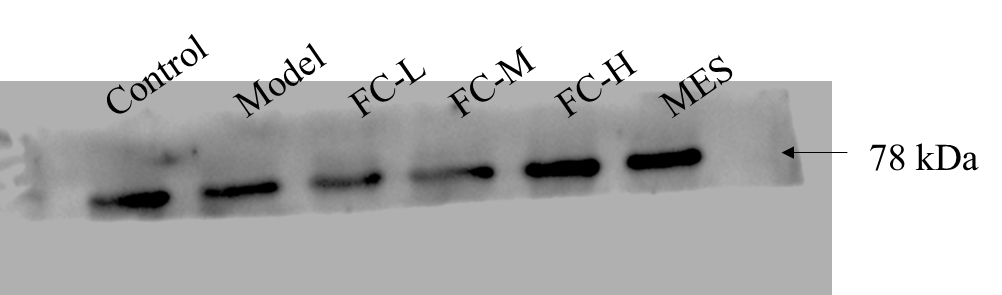
**

**
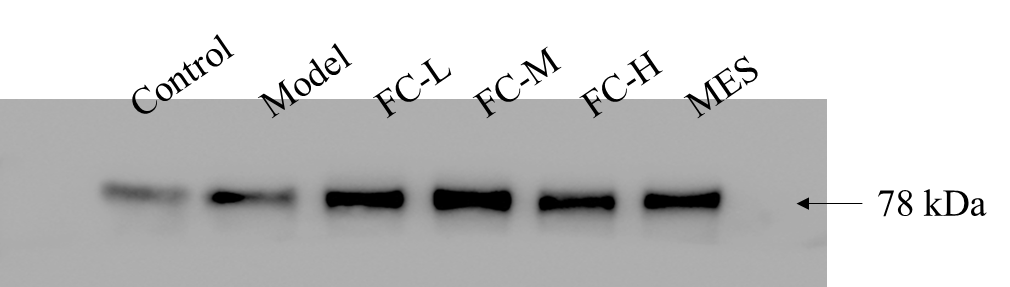
**

**β-actin (42 kDa)**


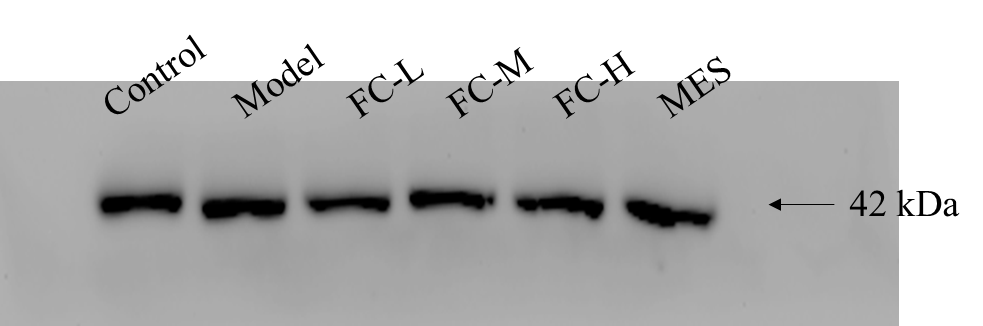


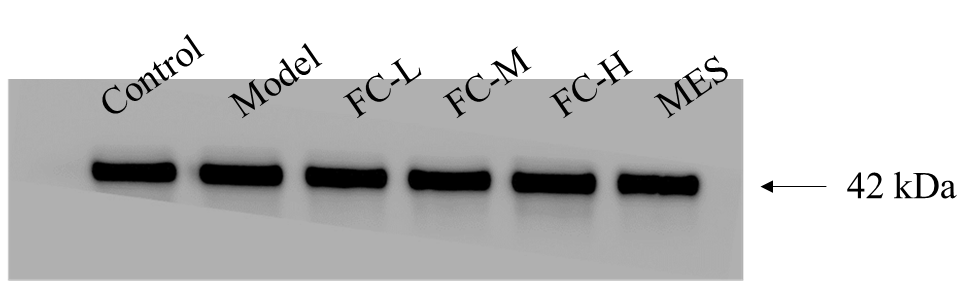


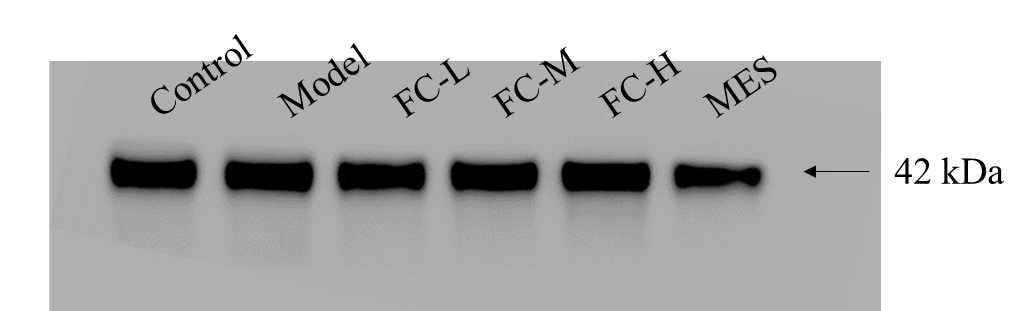

Supplement: Supplementary file 3 — Additional file 3. [file 12906_2023_3983_MOESM3_ESM.docx]
